# Modified DNA vaccine confers improved humoral immune response and effective virus protection against SARS-CoV-2 delta variant

**DOI:** 10.1038/s41598-022-24519-5

**Published:** 2022-12-03

**Authors:** Hiroki Hayashi, Jiao Sun, Yuka Yanagida, Takako Otera, Miwa Sasai, Chin Yang Chang, Jiayu A. Tai, Tomoyuki Nishikawa, Kunihiko Yamashita, Naoki Sakaguchi, Shota Yoshida, Satoshi Baba, Munehisa Shimamura, Sachiko Okamoto, Yasunori Amaishi, Hideto Chono, Junichi Mineno, Hiromi Rakugi, Ryuichi Morishita, Masahiro Yamamoto, Hironori Nakagami

**Affiliations:** 1 Department of Health Development and Medicine, Osaka University Graduate School of Medicine, 2-2 Yamada-Oka, Suita, Osaka 565-0871 Japan; 2 Department of Immunoparasitology, Research Institute for Microbial Diseases, Osaka University, Osaka, Japan; 3 Laboratory of Immunoparasitology, WPI Immunology Frontier Research Center, Osaka University, Osaka, Japan; 4 Department of Device Application for Molecular Therapeutics, Osaka University Graduate School of Medicine, Osaka, Japan; 5 Department of Geriatric and General Medicine, Osaka University Graduate School of Medicine, Osaka, Japan; 6 Department of Neurology, Osaka University Graduate School of Medicine, Osaka, Japan; 7 Department of Clinical Gene Therapy, Osaka University Graduate School of Medicine, Osaka, Japan; 8 Division of Microbiology and Immunology, Center for Infectious Disease Education and Research, Osaka University, Osaka, Japan; 9 Anges Inc., Tokyo, Japan; 10 Daicel Co., Osaka, Japan; 11 Takara Bio Inc., Shiga, Japan

**Keywords:** Infection, Vaccines, DNA vaccines

## Abstract

Coronavirus disease 2019 (COVID-19), caused by severe acute respiratory syndrome coronavirus 2 (SARS-CoV-2), has led to a global pandemic. New technologies have been utilized to develop several types of vaccines to prevent the spread of SARS-CoV-2 infection, including mRNA vaccines. Our group previously developed an effective DNA-based vaccine. However, emerging SARS-CoV-2 variants of concern (VOCs), such as the delta variant, have escaped mutations against vaccine-induced neutralizing antibodies. This suggests that modified vaccines accommodating VOCs need to be developed promptly. Here, we first modified the current DNA vaccine to enhance antigenicity. Compared with the parental DNA vaccine, the modified version (GP∆-DNA vaccine) induced rapid antibody production. Next, we updated the GP∆-DNA vaccine to spike glycoprotein of the delta variant (GP∆-delta DNA vaccine) and compared the efficacy of different injection routes, namely intramuscular injection using a needle and syringe and intradermal injection using a pyro-drive jet injector (PJI). We found that the levels of neutralizing antibodies induced by the intradermal PJI injection were higher than intramuscular injection. Furthermore, the PJI-injected GP∆-delta DNA vaccine effectively protected human angiotensin-converting enzyme 2 (hACE2) knock-in mice from delta-variant infection. These results indicate that the improved DNA vaccine was effective against emerging VOCs and was a potential DNA vaccine platform for future VOCs or global pandemics.

## Introduction

Since coronavirus disease 2019 (COVID-19) became a global pandemic^1,2^, many types of vaccines have been developed using both classical and modern platforms. These vaccines include mRNA-based vaccines and have been approved in many countries^3–5^. Nucleotide-based vaccines can be developed quickly because they can be synthesized using only the genetic information of the virus^6^. Messenger RNA (mRNA) vaccines such as BNT162b2 from Pfizer/BioNTech, mRNA-1273 form Moderna have been approved with high protective efficacy for COVID19^7,8^. Currently those vaccines have been approved and used to combat COVID19 pandemic in many countries. Despites those advantage of high efficacy, one of limitations of mRNA vaccines is requirement of cold or ultracold conditioned storage because of its instability^9^. The distribution of mRNA vaccines is sometimes inadequate due to lacking of cold chain infrastructure, poor environment conditions such as extreme climates, high temperatures^10^. Whereas, DNA-based vaccines is known to be more temperature stable than conventional vaccines with long shelf life, for up to at least 3 months at 30 °C^11,12^, which is one of advantage of DNA vaccine, enabling people living in inaccessible locations to get vaccines. It is important for human family to develop vaccines based on various platforms, classical and novel technologies, to deal with various difficult situations and environments for achievement of worldwide vaccination to fight pandemics.

We previously developed a DNA-based vaccine targeting the severe acute respiratory syndrome coronavirus 2 (SARS-CoV-2) spike^13^, which is commonly targeted by approved and developing vaccines^14^. However, several SARS-CoV-2 variants of concern (VOCs) have emerged with some mutations in the spike, showing a drastic reduction in vaccine- or infection-induced neutralization activity^15–20^. One of the VOCs, the delta variant (B.617.2), possesses T19R, G142D, E156G, 157–158 deletion, L452R, T478K, D614G, P681R, and D950N in the spike. L452R in the receptor-binding domain (RBD) has been shown to increase infectivity and escape vaccine-induced cellular immune response^21–23^. Additionally, P681R strengthens fusion activity, leading to worsened pathogenicity^24^. The effectiveness of the current vaccines targeting ancestral spikes is mostly limited, suggesting that the existing vaccines require modification to increase their efficacy against VOCs.

We previously developed a DNA-based vaccine for the ancestral SARS-CoV-2 strain. Intramuscular administration of a DNA plasmid encoding ancestral spike sequences effectively induced neutralizing antibodies (humoral immunity) and spike-specific T cell activation (cellular immunity), leading to protection against SARS-CoV-2 infection in animal model strains^13^. Furthermore, we developed a pyrodrive jet injector (PJI) to achieve effective intradermal injection of the plasmid DNA and small therapeutic molecules^25,26^. We found that intradermal administration of the DNA vaccine via PJI effectively induced immune responses and viral protection in animal models^27^. Other groups have developed DNA vaccines with intradermal injectors^28–32^.

Coronavirus diseases such as COVID-19 and MERS have historically been combatted through vaccine development^33–35^, however, there is still no approved vaccine for MERS^36^. The spike protein of these viruses is critical for entry into host cells^37^ and has been investigated to enhance vaccine efficacy^38–42^. The C-terminus of the spike contains an intercellular targeting signal that helps induce virus particle formation in beta-coronaviruses, such as SARS-CoV-2 and MERS-CoV^41^. Moreover, mutated or C-terminal deletions of SARS-CoV-2 spike proteins have been shown to enhance cell surface localization^38^. Two proline substitutions at the ectodomain of the spike stabilize the pre-fusion structure by stabilizing the spike protein structure^39,40,42^, suggesting that stabilized antigen expression on the cell surface improves efficacy of the DNA vaccine.

In this study, we developed a DNA vaccine by inducing mutations that improve antigen expression. Additionally, we modified our previous DNA vaccine for improved efficacy against the SARS-CoV-2 delta variant. Furthermore, we compared intramuscular and intradermal injections via PJI to find a more effective vaccine administration route. Finally, we evaluated the efficacy of the delta-adapted DNA vaccine against viral infection in mice.

## Results

### GP∆-DNA vaccine improved antibody production

**Figure 1:**
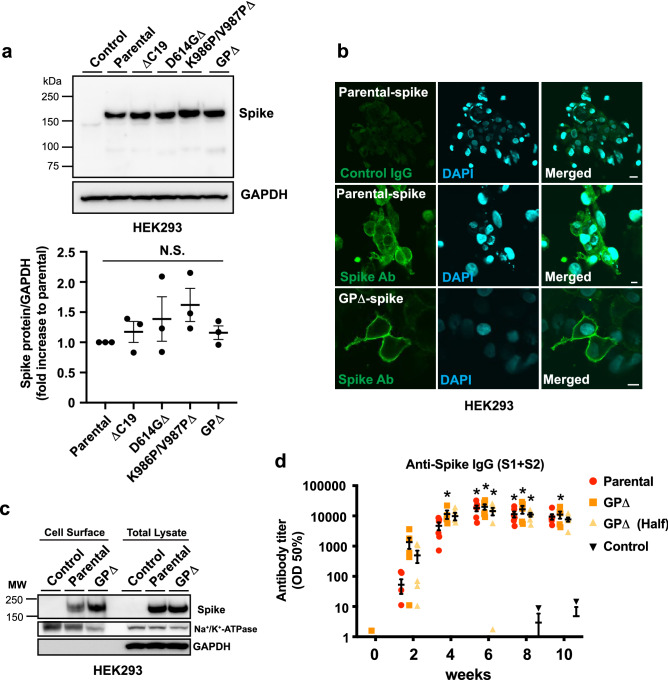
Development of modified DNA vaccine construct. (**a**) Expression of each or all mutated spike glycoprotein in HEK293 cells evaluated through western blotting. Spike was detected using polyclonal SARS-CoV-2 spike antibody (GTX135356, GeneTex). GAPDH was used as a loading control. Lower graph shows the ratio between spike protein and GAPDH, analyzed by densitometric measurement. (**b**) Localization of the spike glycoprotein expressed by the parental DNA vaccine construct, or the GP∆-DNA vaccine construct, assessed through immunostaining in HEK293 cells. Nuclei were stained by DAPI. Scale bars: upper panel (control IgG) = 20 μm, lower 2 panels (anti-Spike Ab) = 10 μm. (**c**) Spike expression on the cell membrane, assessed using cell surface protein isolation. Spike was detected using monoclonal SARS-CoV-2 spike antibody (#42172, Cell Signaling Technology). α1-Na^+^/K^+^-ATPase expression was used as a loading control for isolated surface proteins. (**d**) GP∆-DNA vaccine-induced antibody titer determined using ELISA. Parental DNA vaccine (666.6 μg); Parental, GP∆-DNA vaccine (666.6 μg); GP∆, or half amount of GP∆-DNA vaccine (333.3 μg); GP∆ (Half), with alum adjuvant was intramuscularly injected into male SD rats three times at 2-week intervals. Control rats were not treated. The antibody titer was evaluated for 10 weeks after the first vaccination using ELISA. *p < 0.05 vs. control group (Two-way ANOVA with Tukey’s multiple comparison test). Data are shown as mean ± SEM. See also Supplementary Figs. 1–3.

To improve the efficacy of previously developed DNA vaccines targeting the SARS-CoV-2 spike glycoprotein^13^, multiple mutations and deletions were introduced into the current DNA vaccine based on previous findings. Nineteen amino acids at the C-terminus of the spike protein are intracellular targeting signals in coronaviruses^38,41,43^, and position K986/V987 is important for stabilizing the pre-fusion structure, which is expected to increase the antigenicity of the expressed antigen from the DNA vaccine^39,40,42^. Moreover, SARS-CoV-2 with the D614G mutation in spike glycoprotein, commonly introduced in circulating VOCs, exhibits higher infectivity and enhanced replication^44–46^. First, the effects of these mutations on antigen expression were evaluated in vitro. The expression of spike glycoprotein with each mutation (deletion of 19 aa at the C-terminus: ∆C19, ∆C19; D614G mutation: D614G∆, ∆C19; and K986P/V987P mutation: K986P/V987P∆) or all mutations (GP∆) was not altered (Table 1 and Fig. 1a). The expression of spike glycoprotein from the GP∆-DNA and parental DNA vaccines on the cell membrane was observed through immunocytochemical analysis (Fig. 1b). Furthermore, the expression of spike glycoprotein on the cell surface was compared through western blotting using the isolated cell membrane fractions. Expression of the spike glycoprotein by the GP∆-DNA vaccine on the cell membrane was higher than that by the parental DNA vaccine (Fig. 1c). Secreted spike protein was decreased in the supernatant of HEK293 cells transfected with GP∆-DNA vaccine, compared with parental DNA vaccine (Supplementary Fig. 1). Next, GP∆-DNA vaccine-induced antibody production was compared with that induced by the parental DNA vaccine in Sprague Dawley (SD) rats. The GP∆-DNA vaccine (666.6 μg/body) rapidly increased the antibody titer at 4 weeks (spike antibody titer = 11,506, RBD antibody titer = 10,398). However, the parental vaccine (666.6 μg/body) produced lower antibody titers (spike antibody titer = 4727, RBD antibody titer = 2477). Spike or RBD antibody titers induced by parental DNA vaccine were not significantly different from half amount of GP∆-DNA vaccine (333.3 μg/body) (spike antibody titer = 9875, RBD antibody titer = 6866) (Spike antibody titer: Fig. 1d, RBD antibody titer: Supplementary Fig. 2a). IgG subclasses (IgG1, IgG2a, IgG2b, and IgG2c) were also evaluated using ELISA. IgG2b and IgG2a were the main IgG subclasses in the GP∆-DNA vaccine (Supplementary Fig. 2b), which was consistent with the parental DNA vaccine^13^. Furthermore, the antibody titer remained high until 28 weeks after the first vaccination (Supplementary Fig. 2c,d). A GP∆-DNA vaccine-induced SARS-CoV-2-specific cellular immune response was also confirmed through an ELISPOT. There was no difference between the parental DNA vaccine and GP∆-DNA vaccine (Supplementary Fig. 3a,b). These data suggest that the modified DNA vaccine platform and the GP∆-DNA vaccine had potentiated efficacy, such as a quick antibody response.

**Table 1:** The mutations and deletion in modified plasmid DNA.

Plasmid name	19aa C-ter	D614G	K986P/V987P	Codon
Parental DNA vaccine	+	−	−	Optimized
∆C19	Deleted	−	−	Optimized
D614G∆	Deleted	Mutated	−	Optimized
K986P/V987P∆	Deleted	−	Mutated	Optimized
GP∆	Deleted	Mutated	Mutated	Optimized

### Development of modified GP∆-DNA vaccine targeting delta variant

**Figure 2:**
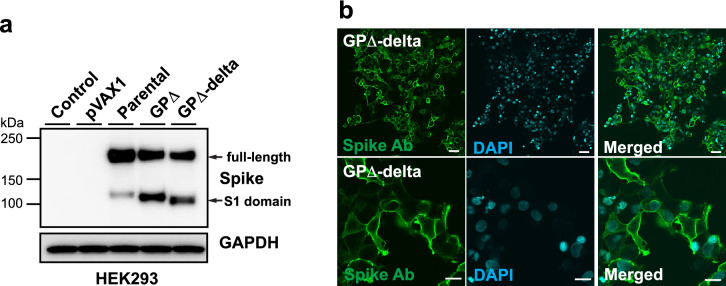
Development of GP∆-DNA vaccine targeting the delta variant. (**a**) Delta variant-specific mutations (Spike mutation of B.1.617.2: T19R, G142D, E156G, del157/158, L452R, T478K, P681R, and D950N) that were induced into the GP∆-DNA vaccine construct. Expression of the delta variant spike was confirmed through western blotting. GAPDH expression was used as a loading control. (**b**) Localization of spike expression in HEK293 cells, assessed through immunocytochemical analysis. Nuclei were stained by DAPI. Scale bars: upper panel = 50 μm, lower panel = 20 μm.

To update the modified DNA vaccine to target the delta variant, mutations in spike glycoprotein of delta variant B.1.617.2 were introduced (T19R, G142D, E156G, del157/158, L452R, T478K, P681R, and D950N) into the plasmid construct of the GP∆-DNA vaccine (GP∆-delta DNA vaccine). The expression of the delta-spike protein from the GP∆-delta DNA vaccine was confirmed using western blotting (Fig. 2a) and immunocytochemical analysis (Fig. 2b).

### Optimization of the administration route and evaluation of the effect of the GP∆-delta DNA vaccine on humoral immune response in rats

**Figure 3:**
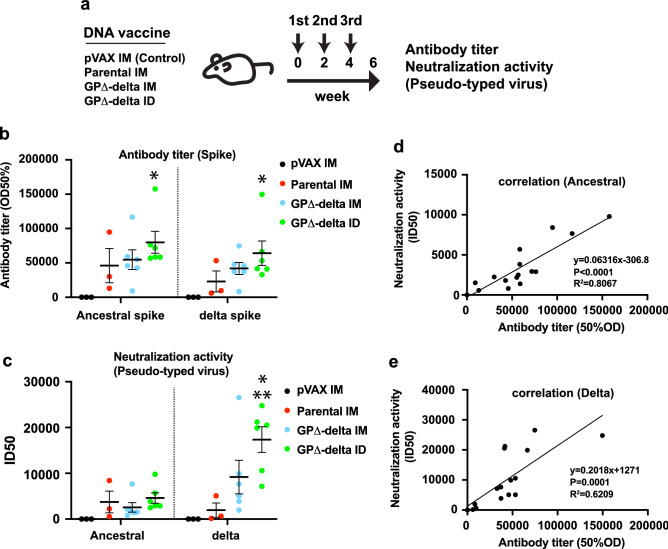
GP∆-delta DNA vaccine induced neutralizing antibodies against delta variant. (**a**) Animal protocol for the control pVAX (intramuscularly; IM), parental DNA (IM), GP∆-delta DNA (IM), or GP∆-delta DNA (intradermally; ID) vaccines using PJI. Control or DNA vaccine was injected three times at 2-week intervals. (**b**) Antibody titers for ancestral and delta spikes, measured using ELISA. *p < 0.01 vs. pVAX1 IM group (Two-way ANOVA with Tukey’s multiple comparison test). (**c**) The neutralizing activity of vaccine-induced antibodies, as determined through VSV-based pseudo-typed viruses carrying the SARS-CoV-2 spike (ancestral or delta variant). The values show the 50% inhibitory dose of serum. *p < 0.01 vs. pVAX1 IM group. **p < 0.05 vs. parental IM group (Two-way ANOVA with Tukey’s multiple comparison test. (**d**) and (**e**) Correlation between ID50 values of immunized serum samples on the pseudo-typed virus and IgG antibody titer for spike. (**d**): Ancestral spike, (**e**): delta variant spike. R^2^, coefficient of determination. Data are shown as mean ± SEM. See also Supplementary Fig. 4.

To compare the reasonable methods of antibody response, intramuscular (IM) and intradermal (ID) injections were evaluated in a rat model. The GP∆-delta DNA vaccine was injected intramuscularly with an alum adjuvant using a needle and syringe or intradermally without an alum adjuvant using a PJI. The parental DNA vaccine was injected intramuscularly with an alum adjuvant as a standard vaccine^13^. The pVAX1 control plasmid was injected intramuscularly with an alum adjuvant as a control (Fig. 3a). The rats were immunized with the vaccine three times at 2-week intervals. Six weeks after the first vaccination, the antibody titer for the ancestral and VOC spikes (B.1.1.7: alpha, B.1.351: beta, P.1: gamma, and B.1.617.2: delta) was determined using ELISA. The anti-delta spike antibody titer in rats immunized with the parental DNA vaccine was 22,976, which is lower than the anti-ancestral spike antibody titer (46,008). Moreover, the antibody titer for the delta spike in the IM GP∆-delta DNA vaccine (41,887) was higher than that of parental DNA vaccine-immunized rats. Intradermal vaccination with a PJI induced the highest antibody titer for both the ancestral and delta spikes (79,984 and 64,141, respectively; Fig. 3b). Similarly, the intradermal GP∆-delta DNA vaccine induced a higher antibody titer for other VOCs (alpha, beta, and gamma; Supplementary Fig. 4). The neutralization activity of the vaccine-induced antibody was evaluated through an in vitro neutralization assay using a vesicular stomatitis virus (VSV) pseudotyped with SARS-CoV-2 spike glycoprotein. The values of neutralization activity for ancestral spike-based, pseudotyped VSVs were almost equal among the groups (ancestral 50% inhibitory dose (ID50): pVAX IM = 5, parental IM = 3740, GP∆-delta IM = 2561, and GP∆-delta ID = 4608). As expected, the intradermal GP∆-delta DNA vaccine-induced antibody exhibited the highest ID50 value for the delta-spike-based pseudotyped VSV (delta variant ID50: pVAX IM = 6, parental IM = 1955, GP∆-delta IM = 9181, and GP∆-delta ID = 17,365; Fig. 3c). Moreover, the antibody titers for ancestral and delta spikes correlated with pseudotyped virus-based neutralization values (ancestral strain: Fig. 3d, delta strain: Fig. 3e). These results suggest that the GP∆-delta DNA vaccine effectively induced neutralizing antibodies against the delta variant, and that intradermal administration by PJI induced a more robust humoral antibody response than intramuscular injection with a needle and syringe.

### PJI-driven intradermal GP∆-delta DNA vaccine protects mice from SARS-CoV-2 delta variant infection in mSP-C hACE2 KI mice

**Figure 4:**
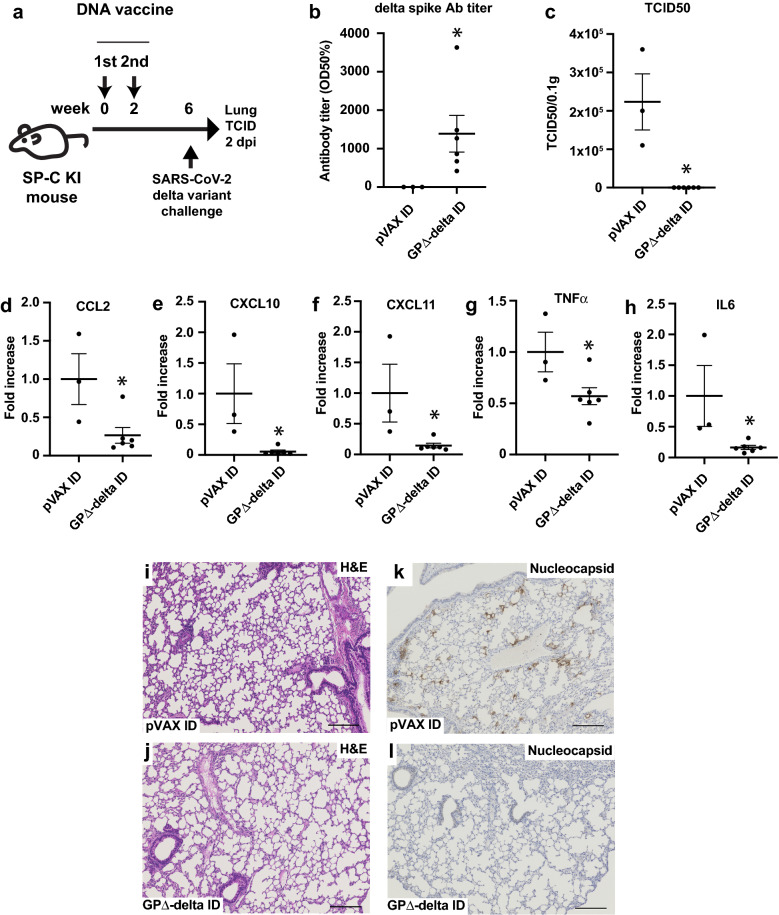
Intradermal administration of GP∆-delta DNA vaccine protects mice from SARS-CoV-2 delta variant infection. (**a**) Animal protocol for intradermal administration of the GP∆-delta DNA vaccine in female SP-C KI mice. The GP∆-delta DNA vaccine was intradermally injected through PJI two times at 2-week intervals. Six weeks after the first dose, the SARS-CoV-2 delta strain was challenged to mice via intranasal inoculation. Two days-post infection, the lungs were extracted to determine virus infection using a TCID assay. (**b**) Antibody titer specific for the delta variant spike, as determined through ELISA. *p < 0.05 vs. control pVAX ID group (Student’s t-test). (**c**) The viral load, which was determined through a plaque assay in the lungs at 2 dpi. *p < 0.01 vs. control pVAX ID group (Student’s t-test). (**d–h**) The inflammatory chemokines (**d**) CCL2, (**e**) CXCL10, (**f**) CXCL11 and cytokines (**g**) TNFα, (**h**) IL6 expression in the lungs after SARS-CoV-2 challenge was evaluated using real-time qPCR. The value was normalized through GAPDH expression. *p < 0.05 vs. control pVAX ID group (Student’s t-test). (**i**, **j**) H&E staining of the lung sections at 2 dpi following SARS-CoV-2 infection. Bar = 200 μm. (**i**) control mice, (**j**) vaccinated mice. (**k**, **l**) Immunohistochemical staining with SARS-CoV-2 nucleocapsid antibody, (**k**) control mice, (**l**) vaccinated mice. Bar = 200 μm. Data are shown as mean ± SEM. See also Supplementary Figs. 5–8.

Finally, the effect of the GP∆-delta DNA vaccine on the delta-variant infection was evaluated in mice. To achieve hACE2 expression in the lungs in vivo, we generated mice possessing human ACE2 in the mouse *SP-C* locus using CRISPR/Cas9 genome editing (Supplementary Fig. 5a,b). We obtained a mouse line and evaluated the expression of hACE2 cDNA in the lungs. Breeding of hACE2 KI mice exhibited typical Mendelian inheritance, and no indication of prenatal lethality. The phenotype and behavior of hACE2 KI mice were normal, compared with wild-type mice. In this study, hACE2 cDNA was highly detected in the lungs of hACE2 KI mice, but not in wild-type mice (Supplementary Fig. 5c). Furthermore, SP-C KI mice were intradermally immunized with the GP∆-delta DNA vaccine using PJI. The immunization was performed three times at 2-week intervals. Six weeks after the first vaccination, the mice were challenged with 2 × 10^5^ TCID_50_ of the SARS-CoV-2 delta strain (Fig. 4a). The antibody titer for the delta spike increased (Fig. 4b). Viral load in the lungs was suppressed by the GP∆-delta DNA vaccine, as shown by the tissue culture ID50 (Fig. 4c). Inflammatory cytokines and chemokines are upregulated following SARS-CoV-2 infection^47,48^. Therefore, pro-inflammatory molecules were evaluated using real-time qPCR in the lungs 2 days after the SARS-CoV-2 challenge. CCL2, CXCL10, CXCL11, TNFα, and IL6 expression was suppressed in the vaccinated group (Fig. 4d–h). In contrast, IFNβ1, IFNγ, CXCL2, and CCL5 expression levels tended to decrease, but not significantly (Supplementary Fig. 6a–h). Histological analysis using H&E staining showed accumulation of immune cells in the perivascular region in the control group (Fig. 4i, Supplementary Fig. 7)^49^, but not in the vaccinated mice (Fig. 4j, Supplementary Fig. 7). Similarly, immunological analysis through nucleocapsid staining showed that the nucleocapsid protein was strongly detected in the control group (Fig. 4k, Supplementary Fig. 8), but not in the vaccinated group (Fig. 4l, Supplementary Fig. 8), suggesting that SARS-CoV-2 infection was effectively suppressed by the GP∆-delta DNA vaccine.

## Discussion

In this study, a previously developed DNA vaccine against SARS-CoV-2 spike was improved through mutations and deletions in the spike protein, resulting in enhanced antigen expression on the cell surface. The redeveloped DNA vaccine (GP∆-DNA vaccine) accelerated antibody production without affecting SARS-CoV-2-specific T cell immune response. PJI-driven intradermal administration of the delta variant-adjusted GP∆-DNA vaccine (GP∆-delta DNA vaccine) induced stronger neutralizing antibody production against the delta variant than intramuscular injection. Finally, intradermal injection of the GP∆-delta DNA vaccine effectively protected the mice from delta SARS-CoV-2 infection.

The SARS-CoV-2 spike protein, composed of the S1 and S2 domains, is a critical component of the host cell infection process^50^. As such, many vaccines primarily target the spike protein^14^. We developed an upgraded DNA vaccine wherein the amino acid sequences of spikes were mutated and partially deleted to enhance vaccine efficacy, based on previous findings^38–42^. The cytoplasmic domain at the C-terminus of the spike contains “KxHxx” sequence motifs as an endoplasmic reticulum (ER) retrieval signal. Therefore, deletion of 19 aa in the C-terminus enhances cell surface translocation^38^. Membrane localization of the GP∆-DNA vaccine-driven spike was increased (Fig. 1c) compared with the parental DNA-driven spike protein. However, this increase did not affect total spike expression (Fig. 1a). It is known that the expression of antigens on the cell surface is critical for the activation of immune cells^51,52^. In this study, the GP∆-DNA vaccine promoted antibody production two weeks after the first vaccination, which was quicker compared to the parental DNA vaccine developed in a previous study (Fig. 1d).

We compared antibody production and neutralization activity between intramuscular and PJI-driven intradermal injections. A meta-analysis of 30 clinical studies (total 177,880 participants) was performed to compare intramuscular and intradermal administration of influenza (H1N1, H3N2, and B strains) vaccines to determine their efficacy. These clinical studies revealed that an intradermal dose of 9 µg was not significantly different from an intramuscular dose of 15 µg. In the H1N1 vaccine, the seroprotection rate of the intradermal dose of 15 µg was higher than that of an equal intramuscular dose. This suggests that a lower dose via intradermal injection is a more effective vaccine administration route against infectious diseases^53^. In this study, the DNA vaccine-driven antibody titer and neutralization activity were higher at 240 μg/body of the intradermal dose via PJI than at the intramuscular dose (666.6 μg/body) for rats (Fig. 3). An intradermal dose of 100 µg/body of parental DNA vaccine also induces a humoral immune response when administered through PJI^27^. In case of DNA vaccination, intradermal route may be more effective than intramuscular route because antigen-expressing antigen presenting cells and keratinocytes evokes a multiple of immune responses under the skin^54–57^. These data suggest that a lower dose of intradermally-administered drugs induces an effective immune response compared to intramuscular injection in DNA vaccination. We previously found that PJI-driven intradermal administration effectively delivers plasmid DNA to the nuclei of the cells in intradermal area, leading to antigen expression in the skin, better than needle-mediated intradermal administration^25,27^. That is possible the reason why PJI-driven administration provided better immune response than intramuscular route in this study. However, further studies are needed to evaluate the lowest dose of each administration route (intradermal or intramuscular) required to achieve viral protection.

We designed the improved DNA vaccine platform for SARS-CoV-2 delta variant, one of VOCs, and evaluated its protective function. However, SARS-CoV-2 Omicron variant (B.1.1.529) have emerged in South Africa from November^58^. Currently, SARS-CoV-2 Omicron variants and sublineages are spreading worldwide. Many reports suggested that SARS-CoV-2 Omicron variants exhibit robust escape from neutralizing antibodies elicited by approved vaccines such as mRNA vaccines^59–62^.Those indicates that development of SARS-CoV-2 Omicron-targeted vaccine is urgent. We are currently working on investigation of the effect of DNA vaccine targeting SARS-COV-2 Omicron variant, designed with improved DNA vaccine platform, developed in this study.

Finally, we modified the previous SARS-CoV-2 DNA vaccine with improved humoral immune responses and updated it to prevent delta variant infection. The modified vaccine showed effective induction of a humoral immune response, leading to viral protection via PJI-driven intradermal administration. Our results prove that DNA vaccines can be quickly modified to protect against potential future variants. Furthermore, these results showcase the current improved DNA vaccine platform as a viable option in case of future pandemics.

## Methods

### Synthesis of modified DNA vaccine (GP∆-DNA vaccine)

Optimized DNA sequences of the spike glycoprotein (ancestral stain) were inserted into the pVAX1 plasmid, as previously described^13^. To synthesize a modified DNA vaccine, 19 amino acids at the C-terminus of the spike protein were deleted. Using gene synthesis, aspartic acid at 614, lysine at 986, and valine at 987 were replaced with glycine, proline, and proline, respectively. The modified DNA vaccine targeting the delta variant and carrying spike mutations based on delta variant B.1.617.2 (T19R, G142D, E156G, del157/158, L452R, T478K, P681R, D950N), was also produced through gene synthesis. The sequences of these plasmids were confirmed using DNA sequencing (Supplementary Information).

### Cell culture and DNA transfection

HEK293 cells were maintained in DMEM (Nacalai Tesque) containing 10% fetal bovine serum (FBS, Sigma) and penicillin/streptomycin (Nacalai Tesque). They were incubated at 37 °C in a humidified 5% CO_2_ incubator (PHCbi). Next, transfection of plasmid DNA was performed using Lipofectamine2000 (Invitrogen) according to the manufacturer’s instructions. After transfection, the cells were incubated for 48 h in FBS-supplemented DMEM and used for western blotting or immunocytochemical analysis.

### Western blotting

Cells transfected with the pVAX-SARS-CoV-2 spike glycoprotein were washed and lysed using a RIPA buffer containing a protease inhibitor cocktail (Roche). Cell lysates were sonicated and centrifuged at 20,000×*g* for 5 min at 4 °C. The supernatant was collected and stored at − 80 °C until use. After protein concentration was measured, the boiled protein samples containing Laemmli sample buffer (Bio-Rad) with β-mercaptoethanol were separated on a 4–20% gradient gel. Next, the samples were transferred to a methanol-activated polyvinylidene difluoride (PVDF) membrane. The membrane was blocked using blocking buffer (PBS containing 0.05% Tween 20 and 5% skim milk) for 1 h at RT. It was subsequently incubated with primary antibodies (SARS-CoV-2 spike #42172, Cell Signaling Technology; SARS-CoV-2 Spike GTX135356, GeneTex; and GAPDH (MAB374), Millipore) at 4 °C overnight. The following day, the membrane was washed with PBS containing 0.05% Tween 20 (PBS-T) and incubated with a secondary antibody labeled with horseradish peroxidase (HRP, GE Healthcare) for 1 h at RT. After washing, the membrane was developed using a substrate (Chemi-Lumi One L, Nacalai Tesque). Finally, the signals were detected using the ChemiDoc™ Touch imaging system (Bio-Rad). Spike protein, or GAPDH expression levels were quantified by using Image Lab software (Bio-Rad).

### Cell surface protein isolation

After transfection, HEK293 cells were washed twice using chilled HBSS buffer. The cells were incubated with 0.25 mg/mL Sulfo-NHS-SS-Biotin in ice-cold HBSS at 4 °C for 30 min. The reaction was halted by washing the cells three times using chilled HBSS containing 100 mM glycine. Additionally, cells were harvested and lysed in the presence of a protease inhibitor cocktail (Roche). The cell lysates were homogenized and centrifuged at 20,000×*g* for 12 min at 4 °C. The supernatant was collected as the sample. After protein quantification, adjusted protein samples were incubated with high-capacity streptavidin agarose (Pierce) for 2 h at 4 °C. The agarose beads were washed three times, and the binding proteins were eluted using Laemmli sample buffer (Bio-Rad) containing β-mercaptoethanol at 95 °C for 5 min. The isolated protein samples were analyzed using western blotting. Anti-alpha 1 sodium potassium ATPase antibody (ab7671, Abcam) was used as a marker of the plasma membrane. Moreover, the GAPDH antibody (MAB374, Millipore) was used as a cytosolic marker.

### Immunocytochemistry

The cells were seeded on glass-bottomed dishes (Matsunami Glass). After plasmid transfection, the cells were washed with PBS and fixed with 4% paraformaldehyde. After blocking with PBS containing 5% skim milk for 1 h at RT, the cells were incubated with anti-SARS-CoV-2 spike proteins (BLSN-005P, Beta Lifescience) or control IgG (Thermo Fisher) at 4 °C overnight. The following day, the cells were washed twice with chilled PBS and incubated with a secondary antibody labelled with Alexa Fluor 488 (Molecular Probes) for 1 h at RT. Cell nuclei were stained with DAPI (Roche). The cells were then observed under a confocal microscope (FV10i, Olympus).

### Quantification of secreted spike protein concentration by using enzyme-linked immunosorbent assay (ELISA)

Secreted spike protein in the cell culture supernatant of HEK293 transfected with was quantified by using ELISA kit (ab284402; Abcam), according to manufacturer’s instructions. Briefly, the pre-coated wells were incubated with the supernatant of HEK293 cells with transfected with parental DNA vaccine or GP∆-DNA vaccine for 2.5 h with shaking. After washing, detection antibody was added to the wells, and incubated for 1 h at RT. Washed wells were incubated with horse-radish peroxidase-conjugated streptavidin for 1 h at 45 min. After washing wells, signal was developed with 3,3′-5,5′-tetramethyl benzidine (TMB) solution. After reaction was halted with stop solution, absorbance of the wells were measured at 450 nm using a microplate reader (Bio-Rad). Protein concentration of HEK293 in each well was quantified, and used for normalization.

### Animal protocol and immunization

Male Crl: Caesarean Derived (CD) Sprague Dawley (SD) rats (7-week-old) were purchased from Charles River (Japan) and housed with free access to food and water in a temperature- and light cycle-controlled facility. All rat experiments were approved by the Ethical Committee for Animal Experiments of the Osaka University Graduate School of Medicine and KAC Inc. (Japan). For intramuscular immunization in rats, a dose of 666.6 μg pVAX1 (control), DNA vaccine, GP∆-DNA vaccine, or GP∆-delta DNA vaccine was administered with 66.7 μL alum adjuvant (Adju-Phos adjuvant: InvivoGen). The intramuscular injections were performed three times at 2-week intervals (200 μL/injection × 2 sites/body) using a needle and syringe. For intradermal immunization in rats using PJI (Actranza lab., Type:Rat, provided by Daicel Co.), a dose of 240 μg of GP∆-delta DNA vaccine was intradermally administered using PJI three times at 2 weeks interval (30 μL/injection × 4 sites/body). All studies involving animals are reported in accordance with *ARRIVE* guidelines.

### Antibody titer determination using enzyme-linked immunosorbent assay (ELISA)

Anti-spike IgG antibody titers were evaluated using ELISA^13^. Briefly, 96-well plates were coated with 1 μg/mL recombinant spike (ancestral spike; BLPSN-0986P: Beta Lifescience, SPN-C52H9: Acro Biosystems, alpha spike: SPN-C52H6: Acro Biosystems, beta spike: SPN-C52Hk: Acro Biosystems, gamma spike: SPN-C52Hg, delta spike: SPN-C52He: Acro Biosystems) at 4 °C overnight. The wells were blocked with 5% skim milk-PBS for 2 h at RT and incubated with diluted sera (from 10- to 31250-fold dilution with 5% skim milk-PBS) at 4 °C overnight. Then, the wells were washed using PBS-T and incubated with HRP-conjugated anti-IgG antibodies (rat: NA935, mouse: NA931; GE Healthcare) for 3 h at RT. The washed wells were developed using the peroxidase chromogenic substrate 3,3′-5,5′-tetramethyl benzidine (Sigma) for 30 min at RT. After the reaction was halted using 0.5 N sulfuric acid, the absorbance of the wells was immediately measured at 450 nm using a microplate reader (Bio-Rad). Furthermore, the half-maximum antibody titer of each sample was determined from the highest absorbance in the dilution range (GraphPad Prism 8 software).

### Enzyme-linked ImmunoSpot (ELISPOT) assay

SARS-CoV-2 spike-specific cellular immune responses were determined through the ELISPOT assay as previously described^13^. Briefly, PVDF membrane-bottomed 96-well plates (Millipore) were incubated with an anti-rat interferon γ (IFNγ) antibody at 4 °C overnight. The following day, the wells were washed and blocked using a blocking solution (UCT Biosciences) for 2 h at RT. Splenocytes were extracted from immunized rats and adjusted to 3 × 10^5^ cells/well. Next, splenocytes were stimulated with 1 μg/mL (final concentration) SARS-CoV-2 spike peptide pools (JPT Peptide Technologies) at 37 °C for 48 h. After the wells were washed, the plate was incubated with a biotinylated polyclonal antibody specific for the rat IFNγ antibody at 4 °C for 2 h, diluted with streptavidin-HRP, and conjugated for 1 h at RT. After the wells were washed using PBS-T, the color was developed using the AEC coloring system (UCT Biosciences). The reaction was stopped by washing with demineralized water. Finally, the colored spots of IFNγ were counted using a dissecting microscope (LMD6500, Leica).

### Pseudo-typed virus production

DNA sequences of the SARS-CoV-2 spike gene (ancestral spike: GenBank: QZC47358.1, delta spike: QWK65230.1) were codon-optimized for human cells and inserted into the eukaryotic expression vector pCAGG to generate envelope plasmids. The envelope plasmids were transfected into HEK293T cells using TransIT-LT1 (Mirus Bio) and incubated for 24 h at 37 °C. The cells were infected with VSV∆G-Luc/G, wherein the G envelope was replaced with the reporter luciferase gene, which was pseudo-typed with the VSV-G glycoprotein^63,64^. The virus was absorbed, washed, and incubated for 24 h at 37 °C. The culture supernatant was collected and stored at − 80 °C after the removal of cell debris via centrifugation.

### Pseudo-typed virus neutralization assay

Vero cells (1.5 × 10^4^ cells/well) were seeded in a 96-well plate and incubated at 37 °C overnight. The rat serum samples were inactivated at 56 °C for 30 min and diluted from 10 to 40,960 dilutions with DMEM. Diluted serum samples (60 µL) were mixed with an equal volume of pseudo-typed virus (equivalent to 2.5 × 10^6^ RLU/mL) for 1 h at 37 °C. Then, 100 μL of mixture (serum/pseudo-typed virus) was added to the Vero cells, and the samples were incubated for 24 h at 37 °C. The cells were lysed and activated using the luciferase assay system (Promega), and their luciferase activity was measured using a Synergy LC (Bio Tek). Neutralization activity was analyzed using GraphPad Prism 8. Moreover, RLU reduction (percentage) was calculated as follows:

$$\frac{{1 - ({\text{RLU }}\;{\text{of }}\;{\text{samples }} - {\text{RLU}}\;{\text{of}}\;{\text{pseudo - typed}}\;{\text{virus - only}}\;{\text{wells}})}}{{({\text{RLU}}\;{\text{from}}\;{\text{ medium - only}}\;{\text{wells}})}} \times 100\%$$

The neutralization titer was calculated as 50% inhibitory dilution (ID50).

### Generation of mouse surfactant-associated protein C (mSP-C) promoter human angiotensin-converting enzyme 2 (hACE2) knock-in (KI) mice

The T7-transcribed mSP-C_gRNA product, which was amplified using KOD FX NEO (Toyobo) and primers (mSP-C_gRNA 5′-TTAATACGACTCACTATAGGgagagagaaaccttacaaaaGTTTTAGAGCTAGAAATAGCAAGTTAAAAT-3′), was used to generate mSP-C_gRNA. The MEGAshortscript T7 kit (Life Technologies) was used to generate these gRNAs. Cas9 mRNA was generated through in vitro transcription (IVT) using the mMESSAGE mMACHINE T7 ULTRA kit (Life Technologies). The template was amplified through PCR using pEF6-hCas9-Puro and primers T7Cas9_IVT_F and Cas9_R, and subsequently gel-purified. In addition, the synthesized gRNA and Cas9 mRNA were purified using a MEGAclear kit (Life Technologies). To obtain SP-C hACE2 KI mice, C57BL/6N female mice (6 weeks old) were superovulated and mated with C57BL/6N stud males. Fertilized one-cell-stage embryos were collected from oviducts and injected into the pronuclei or cytoplasm with 100 ng/µL Cas9 mRNA, 50 ng/µL gRNA, and 50 ng/mL of the targeting vector for SP-C hACE2 KI mice. To generate SP-C hACE2 KI mice, a 0.8 kb fragment containing 500 bp fragments of the intron between exon1 and exon1 of the mouse SP-C gene was amplified through PCR using primers (mSP-C_LA_F 5′-gaattCCAAGCATGCTCATGATCCTAAGCGTGATCCTCAGCACCAGGAGG -3′; mSP-C_LA_R 5′-ggatccTTTGTAAGGTTTCTCTCTCTCTCTCTCCTCTCCTCATCTCTCTGGT -3′; mSP-C_RA_F 5′-acgcgtATGGACATGAGTAGCAAAGAGGTCCTGATGGAGAGTCCACCGGT-3′; mSP-C_RA_R 5′-gtcgacTTCCAGAGATTAAACTCGAGCCGCCACATTTGGTGGCAAGCACC　-3′) and cloned in the pBluescript vector. Human ACE2 cDNA was artificially synthesized and obtained from FASMAC. The hACE2 cDNA cassette and bovine albumin polyA signal sequence were ligated with the mSP-C_LA and mSP-C_RA fragments. The resultant targeting vector was gel-purified and injected into embryos along with mSP-C_gRNA and Cas9 mRNA. Next, injected live embryos were transferred into the oviducts of pseudopregnant ICR females 0.5 d post coitus. The male pup harboring the mutation was mated with C57BL/6N female mice and tested for germline transmission.

To confirm hACE2 mRNA expression in hACE KI mice, RNA was extracted from the kidneys, spleen, and lungs of wild-type (control) or hACE KI mice using QIAzol Lysis reagent (Qiagen) according to the manufacturer’s instructions. Then, cDNA was synthesized from 2 μg total RNA using a High-Capacity cDNA Reverse Transcription Kit (Thermo Fisher). Moreover, hACE2 mRNA expression levels were quantified through quantitative reverse transcription-PCR (QuantStudio6 Pro, Thermo Fisher) using a SYBR green system (Toyobo). The primer sequences were as follows: hACE2_F: CTGGGATCAGAGATCGGAAG, hACE2_R: CAACAGATGGCTGGCAACTA, mouse GAPDH_F: AACAGCAACTCCCACTCTTC, mouse GAPDH_R: CCTGTTGCTGTAGCCGTATT^65^. Mouse GAPDH mRNA expression was used as the control.

### Virus challenge test in mice

Eight- to twelve-week-old female mSP-C- hACE2 KI mice were intradermally vaccinated with the delta DNA vaccine or pVAX1 (control) twice at 2-week intervals. A total of 160 μg (20 μL of 2 mg/mL DNA vaccine/shot, 4 shots/body) of the vaccine was administered using a PJI (Actranza lab., Type:Mouse, Daicel Co.). Six weeks after the first vaccination, mice were intranasally challenged with 2 × 10^5^ TCID_50_ SARS-CoV-2 delta strain (hCoV-19/Japan/TY11-927/2021) under ketamine/xylazine anesthesia. Two days after the viral infection, the lungs were extracted for further evaluation. Animal protocols for the viral challenge were approved by the Animal Research Committee of the Research Institute for Microbial Diseases of Osaka University.

### Virus tissue culture infectious dose determination (TCID_50_)

Virus number in the lungs was determined using the 50% tissue culture infective dose (TCID50). Lung tissues were isolated from each infected mouse at 2 days post-infection (dpi) and homogenized (10 ×) with 100 µL PBS using a disposable homogenizer (BioMasher). Homogenates were centrifuged at 13,000 rpm for 20 min at 4 °C and supernatants were collected in new tubes. The supernatants of lung homogenates were inoculated onto Vero E6 cells that express TMPRSS2 (VeroE6/TMPRSS2) cells in 96-well plates after tenfold serial dilution with DMEM containing 2% FBS. Cells were cultured at 37 °C for 72 h after inoculation, then fixed with 10% formalin.

### Extraction of mRNA and quantitative PCR (qPCR)

Total RNA was isolated from the lungs using TRIzol (Invitrogen) according to the manufacturer’s instructions. Total RNA (2 μg) was used as a template for cDNA conversion using a high-capacity cDNA reverse transcription kit (Thermo Fisher). The synthesized cDNA was used to quantify the target genes through the QuantStudio6 Pro qPCR system (Thermo Fisher) using the following TaqMan primers: IFNα1:Mm03030145_gH, IFNα4: Mm00833969_s1, IFNβ1: Mm00439546_s1, IFNγ: Mm01168134_m1, IL1β: Mm9999906_mH, IL6: Mm00446190_m1, ICAM1: Mm00516025_g1, CCL2: Mm00441243_g1, CXCL2: Mm00436450_m1, TNF: Mm99999068_m1, GAPDH: Mm99999915_g1, CCL5: Mm01302427_m1, CXCL10: Mm00445235_m1, and CXCL11: Mm01345187_g1. Each value was normalized to that of GAPDH.

### Histology and immunohistochemistry

Tissue sections of the lungs collected at 2 dpi were made from 4% paraformaldehyde-fixed, paraffin-embedded samples. The tissue sections were stained with hematoxylin and eosin (H&E), performed by Applied Biomedical Research.

For immunohistochemical analysis, the deparaffinized sections were incubated at 121 °C for 10 min for antigen retrieval. After endogenous peroxidase activity was inactivated with 3% H_2_O_2_, the lung sections were incubated with 10% normal goat serum for 30 min at RT for blocking. The slides were incubated with an anti-SARS-CoV-2 nucleocapsid antibody (40143-R019, Sino Biologicals) for 1 h at RT. After washing, the sections were incubated with HRP-labelled polymer anti-rabbit (Dako Envision system, Dako) for 30 min at RT. HRP signals were developed through incubation using the ImmPACT DAB system (Vector) for 5 min. After the developing reaction was stopped by washing with water, the sections were counterstained with Mayer’s hematoxylin. The mounted sections were observed under a microscope (BZ-X810; Keyence).

### Statistical analysis

All values are presented as the mean ± SEM. To assess significant differences, Student’s *t* test, as well as one- and two-way ANOVA were utilized. These were followed by Tukey’s, or Bonferroni’s multiple comparison test using GraphPad Prism 8 software. Finally, p < 0.05 was considered a statistically significant difference. The method used for statistical analysis was described in each figure legend.

### Statements

All methods in this study were performed in accordance with the relevant guidelines and regulations.

## Supplementary Information

Supplementary Information.

## Data Availability

The datasets used in this study are available from the corresponding author on a reasonable request. Plasmid sequences of GP∆ and GP∆-delta have been deposited into DDBJ/EMBL/GenBank under Accession Numbers LC728279 and LC728280, respectively.
