# Supplementary material for: Modified DNA vaccine confers improved humoral immune response and effective virus protection against SARS-CoV-2 delta variant
Source: Sci Rep. 2022 Dec 3;12:20923. doi: 10.1038/s41598-022-24519-5 (PMC9719526; doi:10.1038/s41598-022-24519-5)
Supplement: Supplementary file 1 — Supplementary Information. [file 41598_2022_24519_MOESM1_ESM.pdf]

Supplementary Fig.1 Secreted spike protein from parental or GPΔ-DNA vaccine.

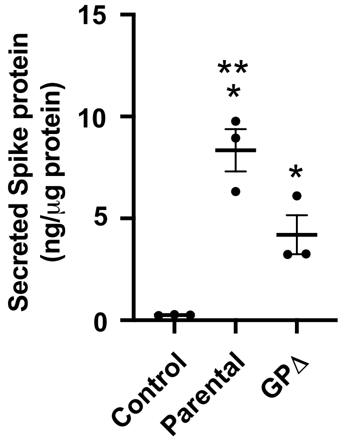

Supplementary Fig.1 Secreted spike protein from parental or GPΔ-DNA vaccine.
Secreted Spike protein in supernatant of HEK293 transfected with parental DNA vaccine or GPΔ-DNA vaccine. . *$p<0.05$ vs.Control, **$p<0.05$ vs.GPΔ (one-way ANOVA with Tukey's multiple comparison test). See also Fig.1.

Supplementary Fig.2 GPΔ-DNA vaccine induced quick antibody production.

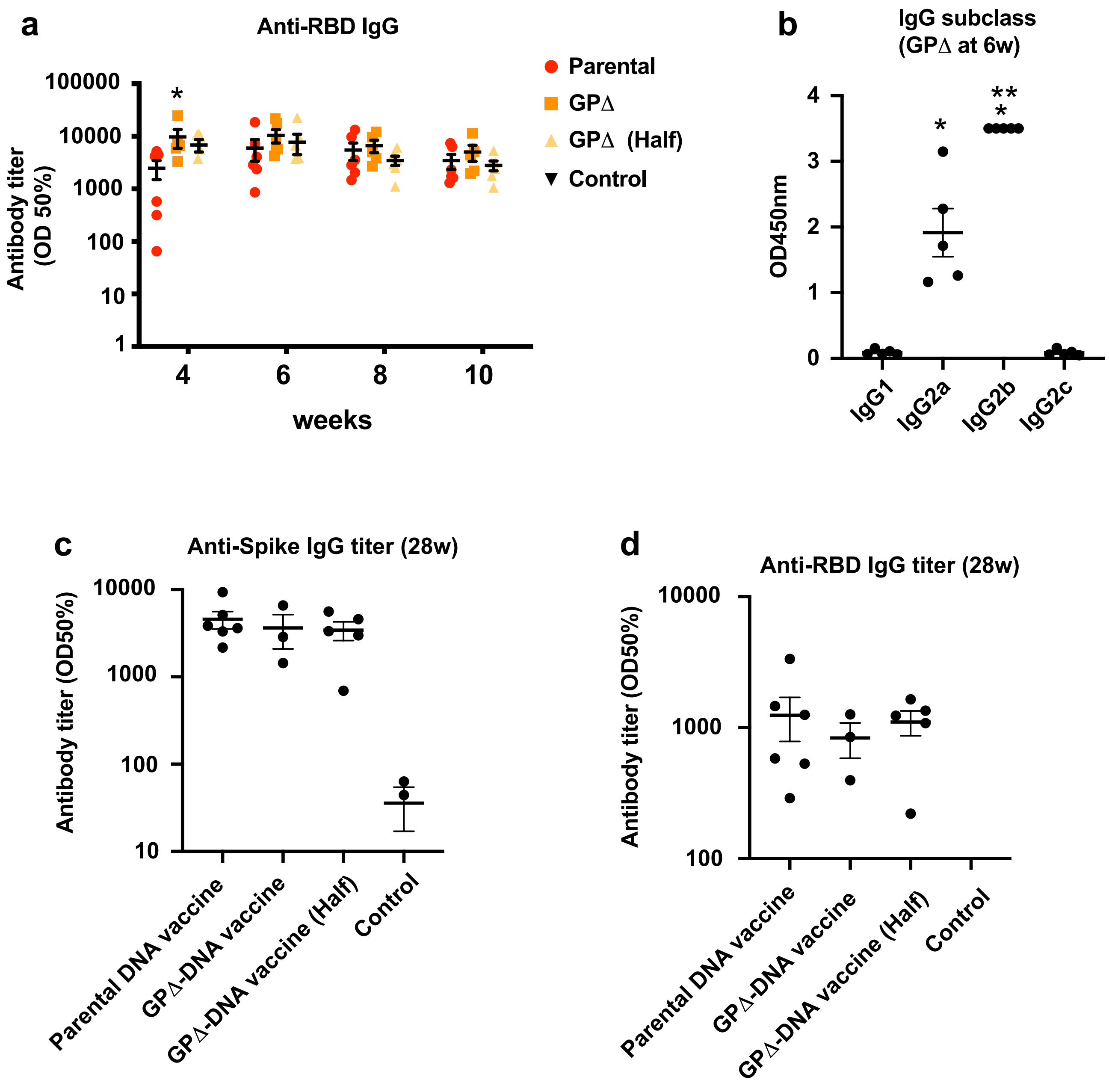

Supplementary Fig.2 GPΔ-DNA vaccine induced quick antibody production.
(a) anti-RBD IgG titer was measured by ELISA from 4 to 10 week after 1st dose of GPΔ-DNA vaccine. Parental DNA vaccine (666.6 μg); Parental, GPΔ-DNA vaccine (666.6 μg); GPΔ or half amount of GPΔ-DNA vaccine (333.3μg); GPΔ (Half), with alum adjuvant was intramuscularly injected into male SD rats three times at 2-week intervals. Control rats were not treated. *p<0.05 vs. Control (two-way ANOVA with Tukey's multiple comparison test). (b) IgG subclass (IgG1, IgG2a, IgG2b, and IgG2c) for spike was analyzed by ELISA. Sera collected at 6 weeks after 1st dose of GPΔ-DNA vaccine was used. *p<0.001 vs. IgG1, IgG2c, **p<0.001 vs. IgG1, IgG2a, respectively (one-way ANOVA with Tukey's multiple comparison test). (c-d) Antiboty titer for spike (c) or RBD (d) was anlyzed at 28 w after 1st dose of GPΔ -DNA vaccine. See also Fig.1.

Supplementary Fig.3 GPΔ-DNA vaccine elicited SARS-CoV-2 specific cellular immune response.

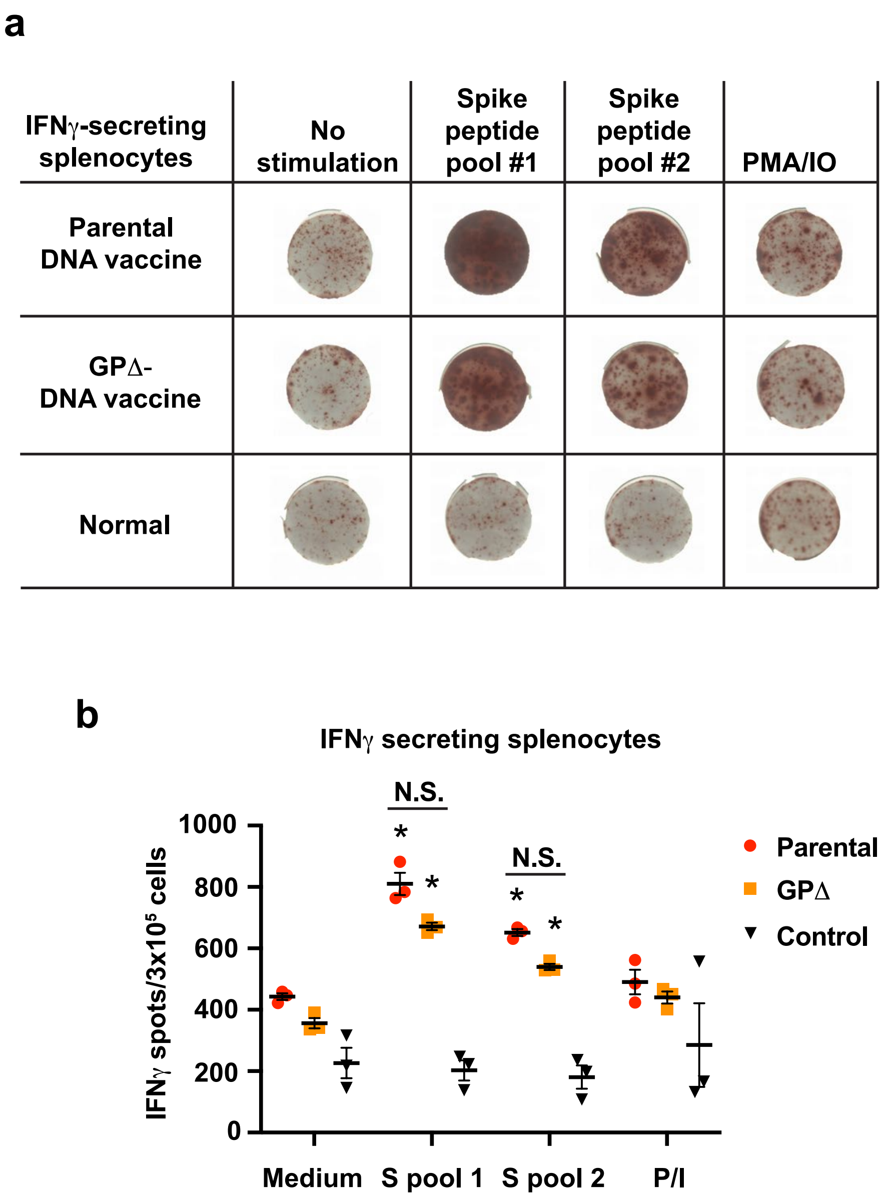

Supplementary Fig.3 GPΔ-DNA vaccine elicited SARS-CoV-2 specific cellular immune response.
(a) SARS-CoV-2 spike-specific IFNγ secreting splenocytes were analyzed by ELISPOT assay. Splenocytes were collected from spleen of immunized rats at 7 weeks after three doses at 2 weeks interval. Splenocytes were stimulated with spike peptide pool for 48 h. (b) IFNγ secreting splenocytes were counted in each wells. *$p<0.001$, vs Control, respectively (two-way ANOVA with Tukey's multiple comparison test). N.S.: not significant between parental and GPΔ group. See also Fig.1.

Supplementary Fig.4 Intradermal GPΔ-delta DNA vaccine induced higher antibody titer for VOCs.

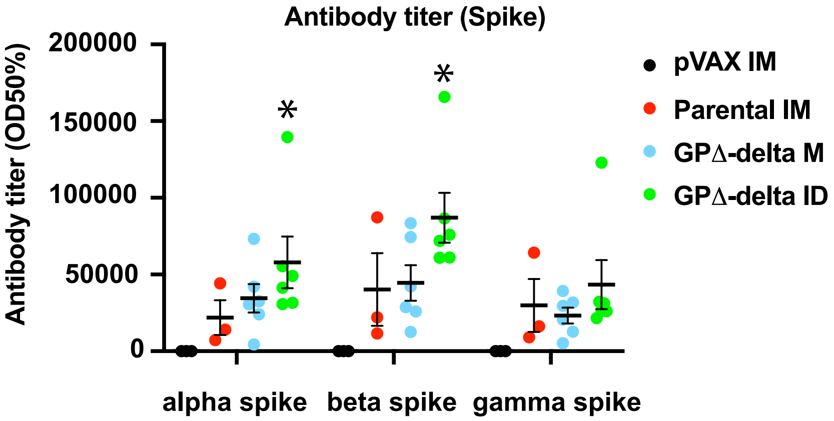

Supplementary Fig.4 Intradermal GPΔ-DNA vaccine-induced antibody titer for VOCs was higher than intramuscular injection.
Antibody titer for VOCs spike (B.1.1.7; alpha, B.1.351; beta, P.1; gamma) was measured by ELISA using serum at 6 weeks. Data are shown as mean ± SEM. *$p<0.01$ vs. pVAX IM (two-way ANOVA with Tukey's multiple comparison test). See also Fig.3.

Supplementary Fig.5 Gereration of mouse SP-C hACE KI mice.

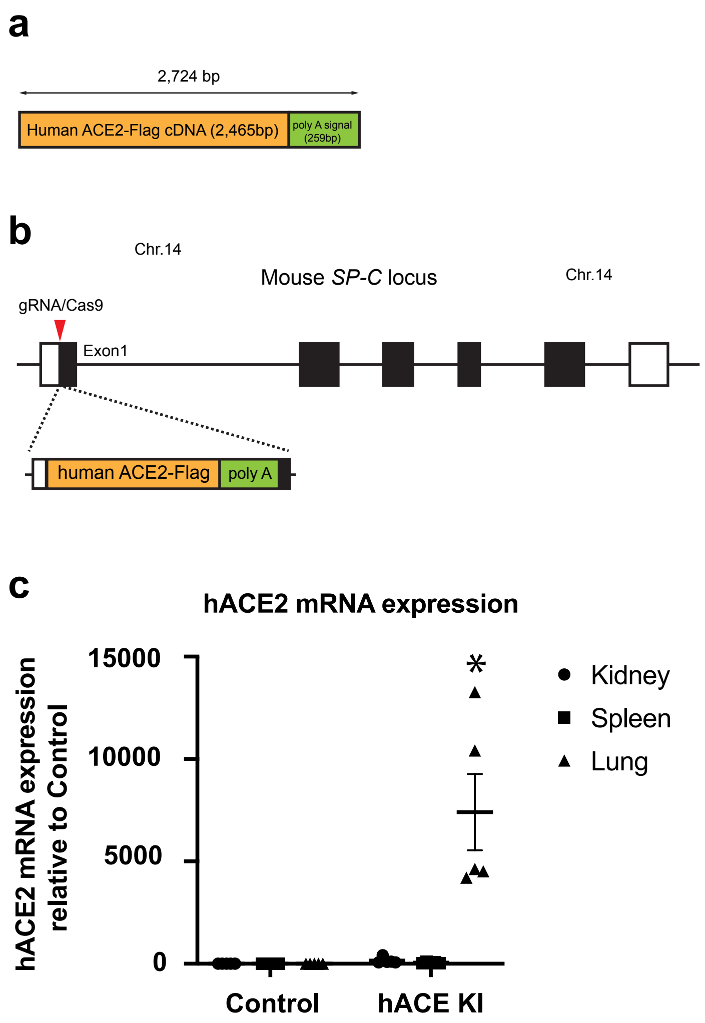

Supplementary Fig.5 Gereration of mouse SP-C hACE KI mice.
(a) Humam ACE2 cDNA and bocine albumin poly A signal sequence used in this study. (b) Targeting strategy for the insertion of hACE2 in the murine *sp-c* locus. (c) Quantitative RT-PCR analysis for detection of human ACE2 mRNA expression in the indicated organs from Control (wild-type) or mouse SP-C hACE2 KI mice. *$p<0.001$ vs. Control (two-way ANOVA with Bonferroni's multiple comparison test). See also Fig.4.

Supplementary Fig.6 The expression profile of cytokines in the lung after SARS-CoV-2 infection.

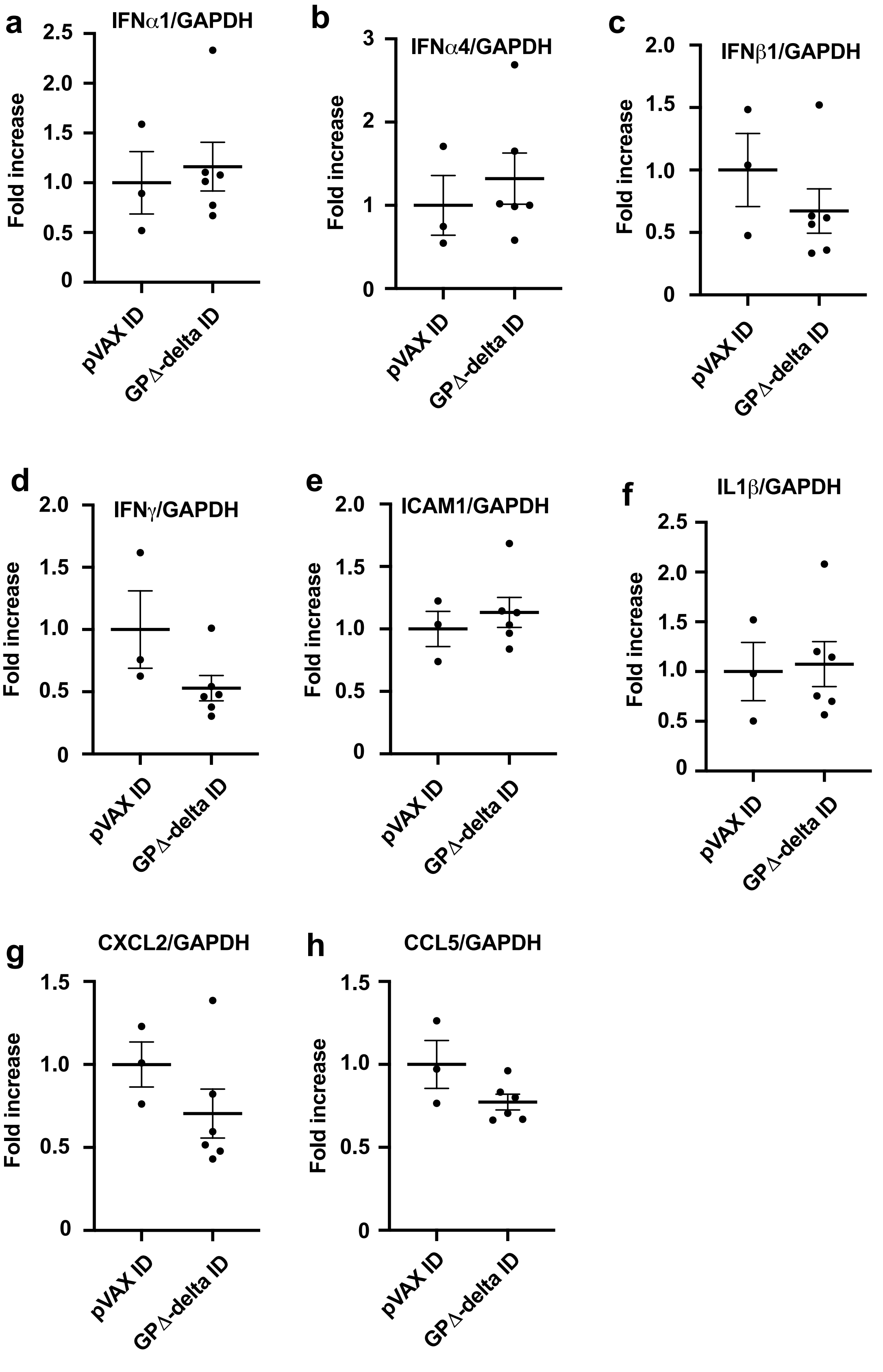

Supplementary Fig.6 The expression profile of cytokines in the lung after SARS-CoV-2 infection.
The cytokines (a) IFNα, (b) IFNα4, (c) IFNβ1, (d) IFNγ, (e) ICAM1, (f) IL1b, (g) CXCL2, (h) CCL5 expression in the lung after SARS-CoV-2 infectino was evaluated by realtime qPCR. The value was normalized with GAPDH. See also Fig.4.

Supplementary Fig.7 Histologial analysis of lung at 2 dpi.

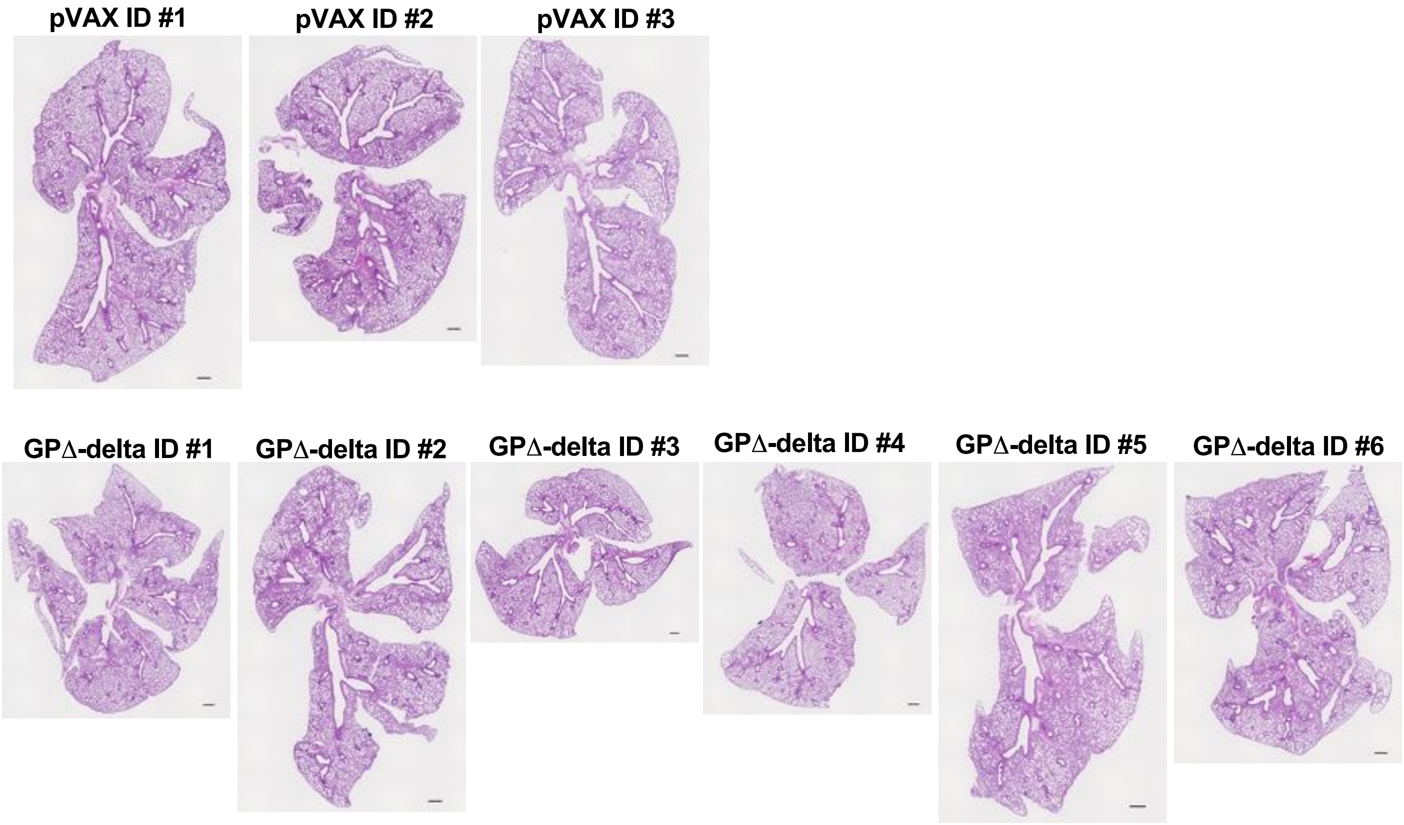

Supplementary Fig.7 Histologial analysis of lung at 2 dpi.
Whole lung image of H&E staining. See also Fig.4.

Supplementary Fig.8 SARS-CoV-2 nucleoprotein expression in the lung 2 dpi.

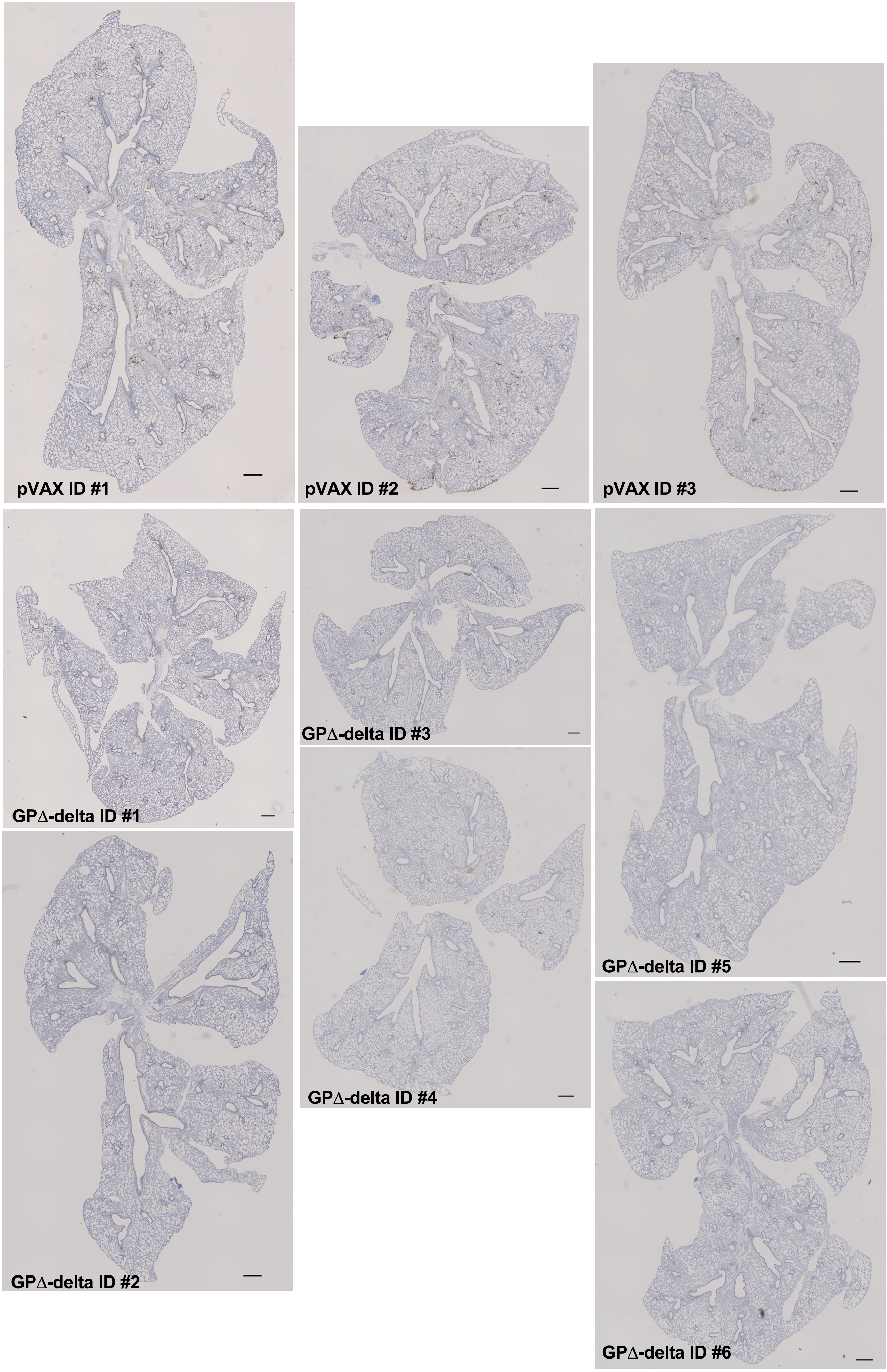

Supplementary Fig.8 SARS-CoV-2 nucleoprotein in the lung 2 dpi.
Whole lung image of SARS-CoV-2 nucleoprotein staining. See also Fig.4.

Supplementary Fig.9 Uncropped western blots related to Fig.1a.

Fig.1a

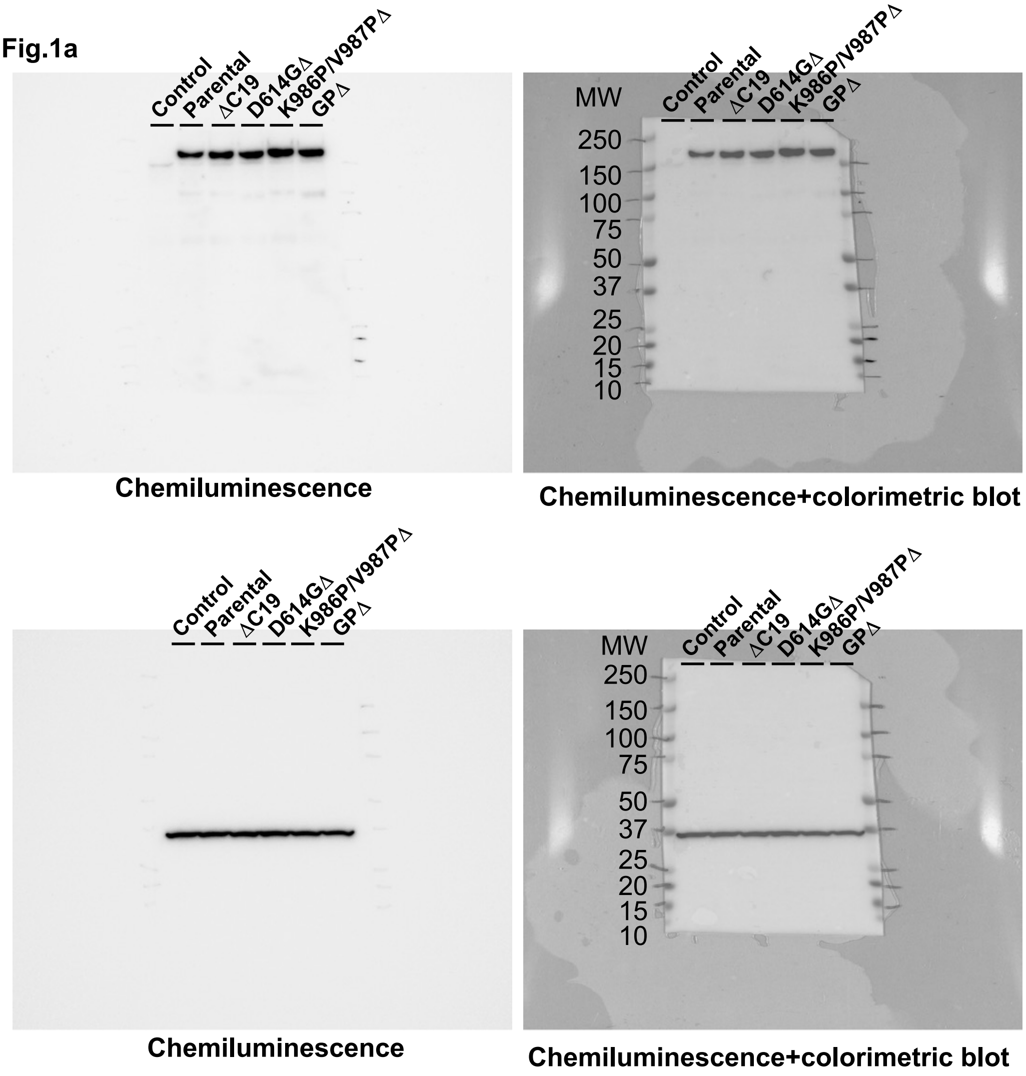

Spike Ab
(GTX135356, GeneTex)

GAPDH
(MAB374, Millipore)

Supplementary Fig.10 Uncropped western blots related to Fig.1c.

Fig.1c

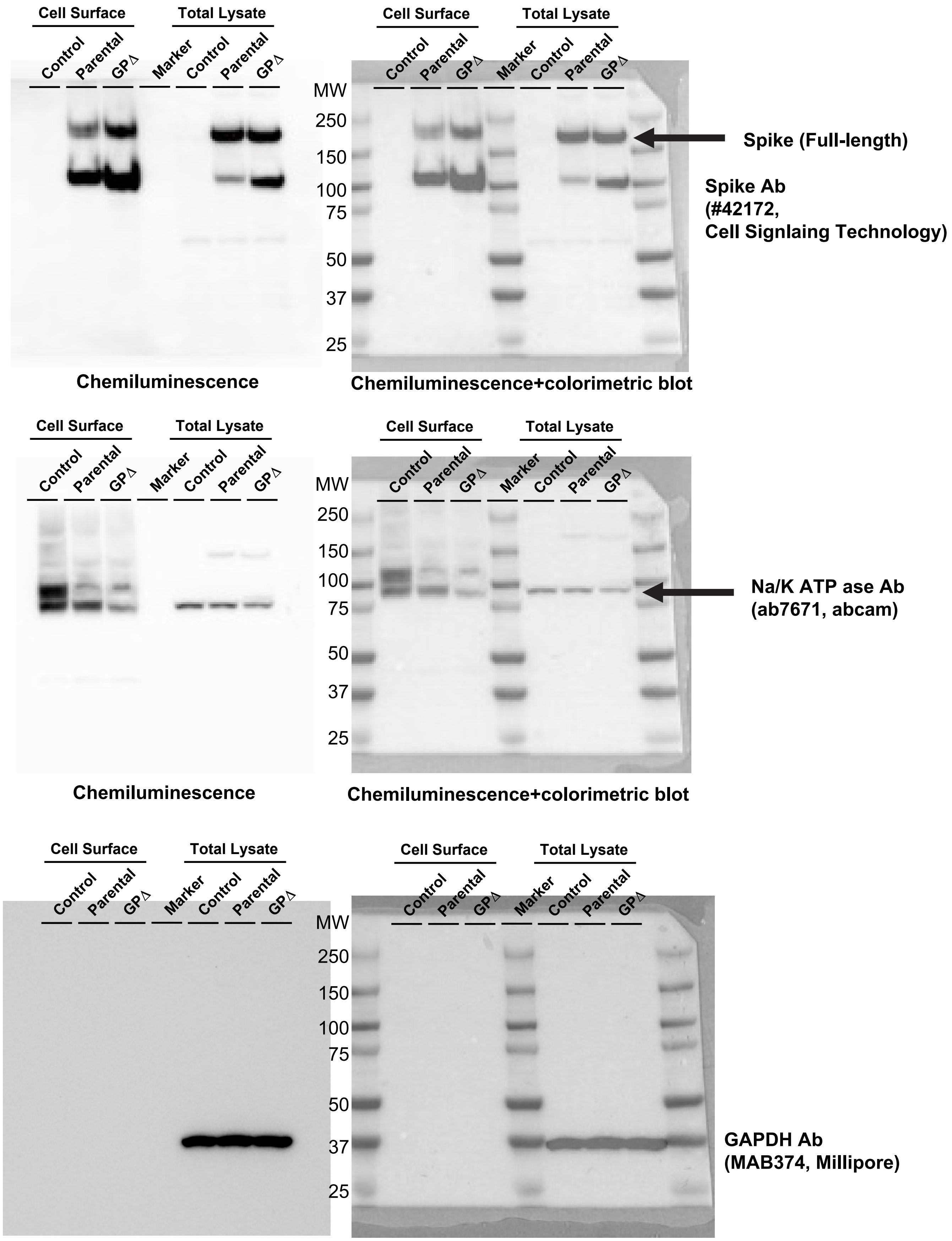

Supplementary Fig.11 Uncropped western blots related to Fig.2a.

Fig.2a

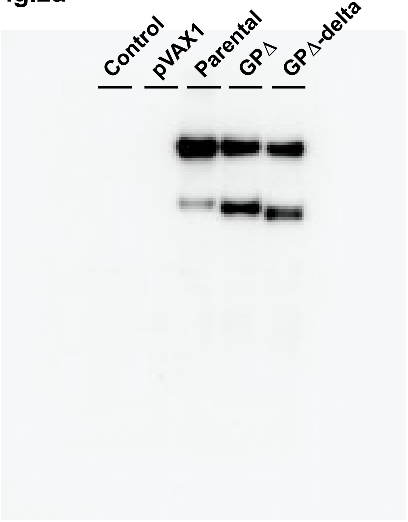

Chemiluminescence

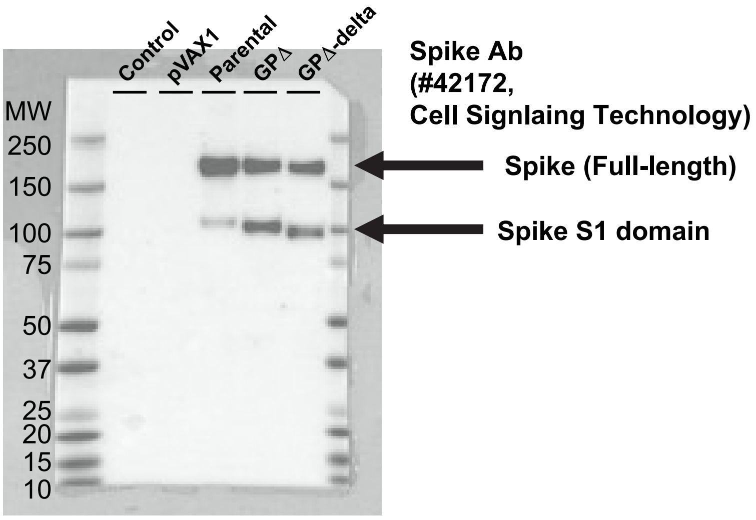

Chemiluminescence+colorimetric blot

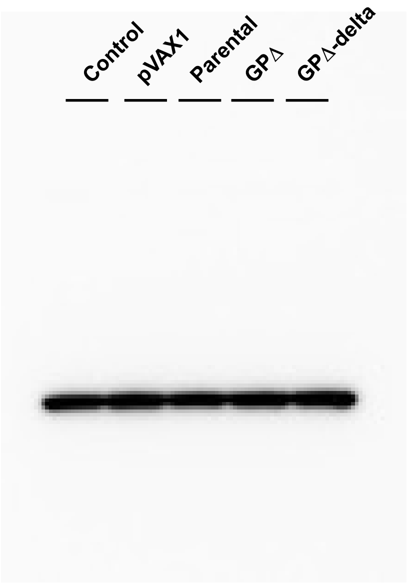

Chemiluminescence

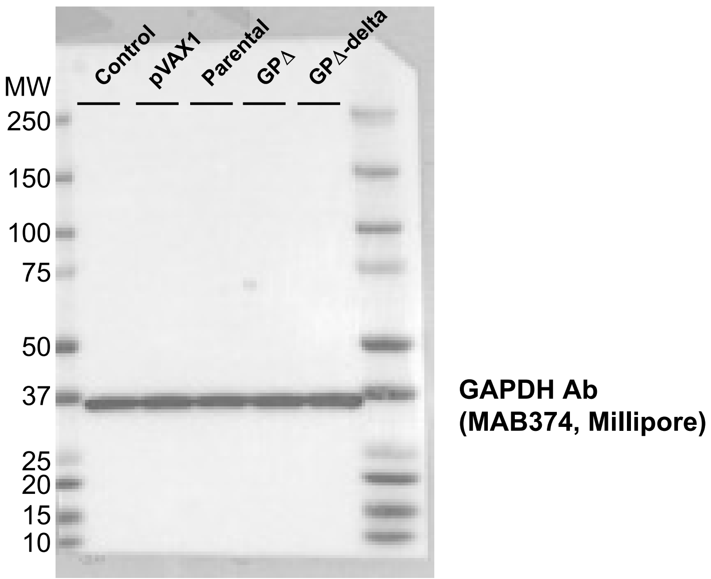

Chemiluminescence+colorimetric blot

Supplementary Information

Sequences of pVAX1-SARS-CoV-2 Spike (GPΔ) plasmid

GCTGCTTCGCGATGTACGGGCCAGATATACGCGTTGACATTGATTATTGACTAGT
TATTAATAGTAATCAATTACGGGGTCATTAGTTCATAGCCCATATATGGAGTTCC
GCGTTACATAACTTACGGTAAATGGCCCGCCTGGCTGACCGCCCAACGACCCCCG
CCCATTGACGTCAATAATGACGTATGTTCCCATAGTAACGCCAATAGGGACTTTC
CATTGACGTCAATGGGTGGAGTATTTACGGTAAACTGCCCACTTGGCAGTACATC
AAGTGTATCATATGCCAAGTACGCCCCCTATTGACGTCAATGACGGTAAATGGCC
CGCCTGGCATTATGCCCAGTACATGACCTTATGGGACTTTCCTACTTGGCAGTAC
ATCTACGTATTAGTCATCGCTATTACCATGGTGATGCGGTTTTGGCAGTACATCA
ATGGGCGTGGATAGCGGTTTGACTCACGGGGATTTCCAAGTCTCCACCCCATTGA
CGTCAATGGGAGTTTGTTTTGGCACCAAAATCAACGGGACTTTCCAAAATGTCGT
AACAACTCCGCCCCATTGACGCAAATGGGCGGTAGGCGTGTACGGTGGGAGGTC
TATATAAGCAGAGCTCTCTGGCTAACTAGAGAACCCACTGCTTACTGGCTTATCG
AAATTAATACGACTCACTATAGGGAGACCCAAGCTGGCTAGCCACCATGTTCGT
GTTCCTGGTGCTGCTGCCCCTGGTGAGCAGCCAGTGCGTGAACCTGACCACCAGA
ACCCAGCTGCCCCCCGCCTACACCAACAGCTTCACCAGAGGCGTGTACTACCCCG
ACAAGGTGTTCAGAAGCAGCGTGCTGCACAGCACCCAGGACCTGTTCCTGCCCTT
CTTCAGCAACGTGACCTGGTTCCACGCCATCCACGTGAGCGGCACCAACGGCAC
CAAGAGATTCGACAACCCCGTGCTGCCCTTCAACGACGGCGTGTACTTCGCCAGC
ACCGAGAAGAGCAACATCATCAGAGGCTGGATCTTCGGCACCACCCTGGACAGC
AAGACCCAGAGCCTGCTGATCGTGAACAACGCCACCAACGTGGTGATCAAGGTG
TGCGAGTTCCAGTTCTGCAACGACCCCTTCCTGGGCGTGTACTACCACAAGAACA
ACAAGAGCTGGATGGAGAGCGAGTTCAGAGTGTACAGCAGCGCCAACAACTGCA
CCTTCGAGTACGTGAGCCAGCCCTTCCTGATGGACCTGGAGGGCAAGCAGGGCA
ACTTCAAGAACCTGAGAGAGTTCGTGTTCAAGAACATCGACGGCTACTTCAAGA
TCTACAGCAAGCACACCCCCATCAACCTGGTGAGAGACCTGCCTCAGGGCTTTAG
CGCCCTGGAGCCACTGGTGGACCTGCCAATCGGCATCAACATCACCAGATTCCA
GACCCTGCTGGCCCTGCACAGAAGCTACCTGACACCAGGCGATTCTAGCTCTGGA
TGGACAGCCGGCGCCGCTGCCTATTACGTGGGCTACCTGCAGCCTAGAACCTTCC
TGCTGAAGTACAACGAGAACGGCACCATCACCGATGCCGTGGACTGCGCCCTGG
ATCCCCTGAGCGAGACCAAGTGTACCCTGAAGAGCTTCACCGTGGAGAAGGGCA
TCTACCAGACCAGCAACTTCAGAGTGCAGCCCACCGAGAGCATCGTGAGATTCC
CCAACATCACCAACCTGTGCCCCTTCGGCGAGGTGTTCAACGCCACCAGATTCGC
CAGCGTGTACGCCTGGAACAGAAAGAGAATCAGCAACTGCGTGGCCGACTACAG
CGTGCTGTACAACAGCGCCAGCTTCAGCACCTTCAAGTGCTACGGCGTGAGCCCC
ACCAAGCTGAACGACCTGTGCTTCACCAACGTGTACGCCGACAGCTTCGTGATCA
GAGGCGACGAGGTGAGACAGATTGCCCCTGGCCAGACCGGCAAGATCGCCGACT
ACAACTACAAGCTGCCCGACGACTTCACCGGCTGCGTGATCGCCTGGAACAGCA
ACAACCTGGACAGCAAGGTGGGCGGCAACTACAACTACCTGTACAGACTGTTCA
GAAAGAGCAACCTGAAGCCCTTCGAGAGAGACATCAGCACCGAGATCTACCAGG
CCGGCTCTACCCCATGCAATGGCGTGGAGGGCTTCAATTGCTACTTCCCCCTGCA
GAGCTACGGCTTCCAGCCCACCAACGGCGTGGGCTACCAGCCCTACAGAGTGGT
GGTGCTGAGCTTTGAACTGCTGCACGCCCCTGCCACCGTGTGCGGCCCAAAGAA
GAGCACCAATCTGGTGAAGAACAAGTGCGTGAACTTCAACTTCAACGGCCTGAC
CGGCACCGGCGTGCTGACCGAGAGCAACAAGAAGTTCCTGCCCTTCCAGCAGTT
CGGCAGAGACATCGCCGACACCACCGATGCCGTGAGAGATCCCCAGACCCTGGA
GATCCTGGACATCACCCCCTGTAGCTTTGGCGGCGTGAGCGTGATTACCCCCGGC

ACCAATACCAGCAACCAGGTGGCCGTGCTGTACCAGGGCGTGAACTGCACCGAG
GTGCCAGTGGCCATCCATGCCGACCAGCTGACCCCAACCTGGAGAGTGTACAGC
ACCGGCAGCAACGTGTTCCAGACAAGAGCCGGCTGTCTGATTGGCGCCGAGCAC
GTGAATAACAGCTACGAGTGCGATATCCCAATCGGCGCCGGCATCTGTGCCAGC
TATCAGACCCAGACCAATAGCCCCAGAAGAGCCAGAAGCGTGGCCAGCCAGAGC
ATCATCGCCTACACCATGAGCCTGGGCGCCGAGAACAGCGTGGCCTACAGCAAC
AACAGCATCGCCATCCCCACCAACTTCACCATCAGCGTGACCACCGAGATCCTGC
CCGTGAGCATGACCAAGACCAGCGTGGACTGCACCATGTACATCTGCGGCGACA
GCACCGAGTGCAGCAACCTGCTGCTGCAGTACGGCAGCTTCTGCACCCAGCTGA
ACAGAGCCCTGACCGGCATCGCCGTGGAGCAGGACAAGAACACCCAGGAGGTGT
TCGCCCAGGTGAAGCAGATCTACAAGACCCCCCCCATCAAGGACTTCGGCGGCT
TCAACTTCAGCCAGATCCTGCCCGACCCCAGCAAGCCCAGCAAGAGAAGCTTCA
TCGAGGACCTGCTGTTCAACAAGGTGACCCTGGCCGACGCCGGCTTCATCAAGC
AGTACGGCGACTGCCTGGGCGACATCGCCGCCAGAGACCTGATCTGCGCCCAGA
AGTTTAATGGACTGACAGTGCTGCCACCCCTGCTGACCGATGAGATGATCGCCCA
GTACACCAGCGCTCTGCTGGCCGGCACAATCACCAGCGGCTGGACATTTGGAGC
CGGAGCCGCTCTGCAGATCCCATTTGCCATGCAGATGGCCTACAGATTCAACGGC
ATCGGCGTGACCCAGAACGTGCTGTACGAGAACCAGAAGCTGATCGCCAACCAG
TTCAACAGCGCCATCGGCAAGATCCAGGACAGCCTGTCTAGCACAGCCTCTGCCC
TGGGCAAGCTGCAGGATGTGGTGAACCAGAACGCCCAGGCCCTGAACACCCTGG
TGAAGCAGCTGAGCAGCAACTTCGGCGCCATCAGCAGCGTGCTGAACGACATCC
TGAGCAGACTGGACCCCCCTGAGGCCGAGGTGCAGATCGACAGACTGATCACCG
GCAGACTGCAGAGCCTGCAGACCTACGTGACCCAGCAGCTGATCAGAGCCGCCG
AAATCAGAGCCAGCGCCAATCTGGCCGCCACCAAGATGAGCGAGTGCGTGCTGG
GCCAGAGCAAGAGAGTGGACTTCTGCGGCAAGGGCTACCACCTGATGAGCTTTC
CCCAGAGCGCCCCTCACGGCGTGGTGTTTCTGCACGTGACCTACGTGCCTGCCCA
GGAGAAGAACTTCACCACCGCCCCTGCCATCTGCCACGATGGCAAGGCCCACTT
CCCTAGAGAGGGCGTGTTCGTGAGCAACGGCACCCACTGGTTCGTGACCCAGAG
AAACTTCTACGAGCCCCAGATCATCACCACCGACAACACCTTCGTGAGCGGCAA
CTGCGACGTGGTGATCGGCATCGTGAACAACACCGTGTACGACCCCCTGCAGCC
CGAGCTGGACAGCTTCAAGGAGGAGCTGGACAAGTACTTCAAGAACCACACCAG
CCCCGATGTGGACCTGGGCGATATCAGCGGCATCAATGCCAGCGTGGTGAACAT
CCAGAAGGAGATCGACCGGCTCAATGAGGTGGCCAAGAACCTGAACGAGAGCCT
GATCGACCTGCAGGAACTGGGCAAATATGAGCAGTACATCAAGTGGCCCTGGTA
CATCTGGCTGGGCTTCATCGCCGGCCTGATCGCCATCGTGATGGTGACCATCATG
CTGTGCTGCATGACCAGCTGCTGCAGCTGCCTGAAGGGCTGCTGCAGCTGCGGGT
CTTGTTGCTAATCTAGAGGGCCCGTTTAAACCCGCTGATCAGCCTCGACTGTGCC
TTCTAGTTGCCAGCCATCTGTTGTTTGCCCCTCCCCCGTGCCTTCCTTGACCCTGG
AAGGTGCCACTCCCACTGTCCTTTCCTAATAAAATGAGGAAATTGCATCGCATTG
TCTGAGTAGGTGTCATTCTATTCTGGGGGGTGGGGTGGGGCAGGACAGCAAGGG
GGAGGATTGGGAAGACAATAGCAGGCATGCTGGGGATGCGGTGGGCTCTATGGC
TTCTACTGGGCGGTTTTATGGACAGCAAGCGAACCGGAATTGCCAGCTGGGGCG
CCCTCTGGTAAGGTTGGGAAGCCCTGCAAAGTAAACTGGATGGCTTTCTTGCCGC
CAAGGATCTGATGGCGCAGGGGATCAAGCTCTGATCAAGAGACAGGATGAGGAT
CGTTTCGCATGATTGAACAAGATGGATTGCACGCAGGTTCTCCGGCCGCTTGGGT
GGAGAGGCTATTCGGCTATGACTGGGCACAACAGACAATCGGCTGCTCTGATGC
CGCCGTGTTCCGGCTGTCAGCGCAGGGGCGCCCGGTTCTTTTTGTCAAGACCGAC
CTGTCCGGTGCCCTGAATGAACTGCAAGACGAGGCAGCGCGGCTATCGTGGCTG
GCCACGACGGGCGTTCCTTGCGCAGCTGTGCTCGACGTTGTCACTGAAGCGGGA
AGGGACTGGCTGCTATTGGGCGAAGTGCCGGGGCAGGATCTCCTGTCATCTCACC

TTGCTCCTGCCGAGAAAGTATCCATCATGGCTGATGCAATGCGGCGGCTGCATAC
GCTTGATCCGGCTACCTGCCCATTCGACCACCAAGCGAAACATCGCATCGAGCG
AGCACGTACTCGGATGGAAGCCGGTCTTGTCGATCAGGATGATCTGGACGAAGA
GCATCAGGGGCTCGCGCCAGCCGAACTGTTCGCCAGGCTCAAGGCGAGCATGCC
CGACGGCGAGGATCTCGTCGTGACCCATGGCGATGCCTGCTTGCCGAATATCATG
GTGGAAAATGGCCGCTTTTCTGGATTCATCGACTGTGGCCGGCTGGGTGTGGCGG
ACCGCTATCAGGACATAGCGTTGGCTACCCGTGATATTGCTGAAGAGCTTGGCGG
CGAATGGGCTGACCGCTTCCTCGTGCTTTACGGTATCGCCGCTCCCGATTCGCAG
CGCATCGCCTTCTATCGCCTTCTTGACGAGTTCTTCTGAATTATTAACGCTTACAA
TTTCCTGATGCGGTATTTTCTCCTTACGCATCTGTGCGGTATTTCACACCGCATCA
GGTGGCACTTTTCGGGGAAATGTGCGCGGAACCCCTATTTGTTTATTTTTCTAAAT
ACATTCAAATATGTATCCGCTCATGAGACAATAACCCTGATAAATGCTTCAATAA
TAGCACGTGCTAAAACTTCATTTTTAATTTAAAAGGATCTAGGTGAAGATCCTTT
TTGATAATCTCATGACCAAAATCCCTTAACGTGAGTTTTCGTTCCACTGAGCGTC
AGACCCCGTAGAAAAGATCAAAGGATCTTCTTGAGATCCTTTTTTTCTGCGCGTA
ATCTGCTGCTTGCAAACAAAAAAACCACCGCTACCAGCGGTGGTTTGTTTGCCGG
ATCAAGAGCTACCAACTCTTTTTCCGAAGGTAACTGGCTTCAGCAGAGCGCAGAT
ACCAAATACTGTTCTTCTAGTGTAGCCGTAGTTAGGCCACCACTTCAAGAACTCT
GTAGCACCGCCTACATACCTCGCTCTGCTAATCCTGTTACCAGTGGCTGCTGCCA
GTGGCGATAAGTCGTGTCTTACCGGGTTGGACTCAAGACGATAGTTACCGGATA
AGGCGCAGCGGTCGGGCTGAACGGGGGGTTCGTGCACACAGCCCAGCTTGGAGC
GAACGACCTACACCGAACTGAGATACCTACAGCGTGAGCTATGAGAAAGCGCCA
CGCTTCCCGAAGGGAGAAAGGCGGACAGGTATCCGGTAAGCGGCAGGGTCGGA
ACAGGAGAGCGCACGAGGGAGCTTCCAGGGGGAAACGCCTGGTATCTTTATAGT
CCTGTCGGGTTTCGCCACCTCTGACTTGAGCGTCGATTTTTGTGATGCTCGTCAGG
GGGGCGGAGCCTATGGAAAAACGCCAGCAACGCGGCCTTTTTACGGTTCCTGGC
CTTTTGCTGGCCTTTTGCTCACATGTTCTT

Sequences of pVAX1-SARS-CoV-2 delta Spike (GPΔ) plasmid

GCTGCTTCGCGATGTACGGGCCAGATATACGCGTTGACATTGATTATTGACTAGT
TATTAATAGTAATCAATTACGGGGTCATTAGTTCATAGCCCATATATGGAGTTCC
GCGTTACATAACTTACGGTAAATGGCCCGCCTGGCTGACCGCCCAACGACCCCCG
CCCATTGACGTCAATAATGACGTATGTTCCCATAGTAACGCCAATAGGGACTTTC
CATTGACGTCAATGGGTGGAGTATTTACGGTAAACTGCCCACTTGGCAGTACATC
AAGTGTATCATATGCCAAGTACGCCCCCTATTGACGTCAATGACGGTAAATGGCC
CGCCTGGCATTATGCCCAGTACATGACCTTATGGGACTTTCCTACTTGGCAGTAC
ATCTACGTATTAGTCATCGCTATTACCATGGTGATGCGGTTTTGGCAGTACATCA
ATGGGCGTGGATAGCGGTTTGACTCACGGGGATTTCCAAGTCTCCACCCCATTGA
CGTCAATGGGAGTTTGTTTTGGCACCAAAATCAACGGGACTTTCCAAAATGTCGT
AACAACTCCGCCCCATTGACGCAAATGGGCGGTAGGCGTGTACGGTGGGAGGTC
TATATAAGCAGAGCTCTCTGGCTAACTAGAGAACCCACTGCTTACTGGCTTATCG
AAATTAATACGACTCACTATAGGGAGACCCAAGCTGGCTAGCCACCATGTTCGT
GTTCCTGGTGCTGCTGCCCCTGGTGAGCAGCCAGTGCGTGAACCTGAGAACCAG
AACCCAGCTGCCCCCCGCCTACACCAACAGCTTCACCAGAGGCGTGTACTACCCC
GACAAGGTGTTCAGAAGCAGCGTGCTGCACAGCACCCAGGACCTGTTCCTGCCC
TTCTTCAGCAACGTGACCTGGTTCCACGCCATCCACGTGAGCGGCACCAACGGCA
CCAAGAGATTCGACAACCCCGTGCTGCCCTTCAACGACGGCGTGTACTTCGCCAG
CACCGAGAAGAGCAACATCATCAGAGGCTGGATCTTCGGCACCACCCTGGACAG
CAAGACCCAGAGCCTGCTGATCGTGAACAACGCCACCAACGTGGTGATCAAGGT

GTGCGAGTTCCAGTTCTGCAACGACCCCTTCCTGGACGTGTACTACCACAAGAAC
AACAAGAGCTGGATGGAGAGCGGCxxxxxxGTGTACAGCAGCGCCAACAACTGCA
CCTTCGAGTACGTGAGCCAGCCCTTCCTGATGGACCTGGAGGGCAAGCAGGGCA
ACTTCAAGAACCTGAGAGAGTTCGTGTTCAAGAACATCGACGGCTACTTCAAGA
TCTACAGCAAGCACACCCCCATCAACCTGGTGAGAGACCTGCCTCAGGGCTTTAG
CGCCCTGGAGCCACTGGTGGACCTGCCAATCGGCATCAACATCACCAGATTCCA
GACCCTGCTGGCCCTGCACAGAAGCTACCTGACACCAGGCGATTCTAGCTCTGGA
TGGACAGCCGGCGCCGCTGCCTATTACGTGGGCTACCTGCAGCCTAGAACCTTCC
TGCTGAAGTACAACGAGAACGGCACCATCACCGATGCCGTGGACTGCGCCCTGG
ATCCCCTGAGCGAGACCAAGTGTACCCTGAAGAGCTTCACCGTGGAGAAGGGCA
TCTACCAGACCAGCAACTTCAGAGTGCAGCCCACCGAGAGCATCGTGAGATTCC
CCAACATCACCAACCTGTGCCCCTTCGGCGAGGTGTTCAACGCCACCAGATTCGC
CAGCGTGTACGCCTGGAACAGAAAGAGAATCAGCAACTGCGTGGCCGACTACAG
CGTGCTGTACAACAGCGCCAGCTTCAGCACCTTCAAGTGCTACGGCGTGAGCCCC
ACCAAGCTGAACGACCTGTGCTTCACCAACGTGTACGCCGACAGCTTCGTGATCA
GAGGCGACGAGGTGAGACAGATTGCCCCTGGCCAGACCGGCAAGATCGCCGACT
ACAACTACAAGCTGCCCGACGACTTCACCGGCTGCGTGATCGCCTGGAACAGCA
ACAACCTGGACAGCAAGGTGGGCGGCAACTACAACTACAGATACAGACTGTTCA
GAAAGAGCAACCTGAAGCCCTTCGAGAGAGACATCAGCACCGAGATCTACCAGG
CCGGCTCTAAGCCATGCAATGGCGTGGAGGGCTTCAATTGCTACTTCCCCCTGCA
GAGCTACGGCTTCCAGCCCACCAACGGCGTGGGCTACCAGCCCTACAGAGTGGT
GGTGCTGAGCTTTGAACTGCTGCACGCCCCTGCCACCGTGTGCGGCCCAAAGAA
GAGCACCAATCTGGTGAAGAACAAGTGCGTGAACTTCAACTTCAACGGCCTGAC
CGGCACCGGCGTGCTGACCGAGAGCAACAAGAAGTTCCTGCCCTTCCAGCAGTT
CGGCAGAGACATCGCCGACACCACCGATGCCGTGAGAGATCCCCAGACCCTGGA
GATCCTGGACATCACCCCCTGTAGCTTTGGCGGCGTGAGCGTGATTACCCCCGGC
ACCAATACCAGCAACCAGGTGGCCGTGCTGTACCAGGGCGTGAACTGCACCGAG
GTGCCAGTGGCCATCCATGCCGACCAGCTGACCCCAACCTGGAGAGTGTACAGC
ACCGGCAGCAACGTGTTCCAGACAAGAGCCGGCTGTCTGATTGGCGCCGAGCAC
GTGAATAACAGCTACGAGTGCGATATCCCAATCGGCGCCGGCATCTGTGCCAGC
TATCAGACCCAGACCAATAGCAGAAGAAGAGCCAGAAGCGTGGCCAGCCAGAG
CATCATCGCCTACACCATGAGCCTGGGCGCCGAGAACAGCGTGGCCTACAGCAA
CAACAGCATCGCCATCCCCACCAACTTCACCATCAGCGTGACCACCGAGATCCTG
CCCGTGAGCATGACCAAGACCAGCGTGGACTGCACCATGTACATCTGCGGCGAC
AGCACCGAGTGCAGCAACCTGCTGCTGCAGTACGGCAGCTTCTGCACCCAGCTG
AACAGAGCCCTGACCGGCATCGCCGTGGAGCAGGACAAGAACACCCAGGAGGT
GTTCGCCCAGGTGAAGCAGATCTACAAGACCCCCCCCATCAAGGACTTCGGCGG
CTTCAACTTCAGCCAGATCCTGCCCGACCCCAGCAAGCCCAGCAAGAGAAGCTT
CATCGAGGACCTGCTGTTCAACAAGGTGACCCTGGCCGACGCCGGCTTCATCAA
GCAGTACGGCGACTGCCTGGGCGACATCGCCGCCAGAGACCTGATCTGCGCCCA
GAAGTTTAATGGACTGACAGTGCTGCCACCCCTGCTGACCGATGAGATGATCGCC
CAGTACACCAGCGCTCTGCTGGCCGGCACAATCACCAGCGGCTGGACATTTGGA
GCCGGAGCCGCTCTGCAGATCCCATTTGCCATGCAGATGGCCTACAGATTCAACG
GCATCGGCGTGACCCAGAACGTGCTGTACGAGAACCAGAAGCTGATCGCCAACC
AGTTCAACAGCGCCATCGGCAAGATCCAGGACAGCCTGTCTAGCACAGCCTCTG
CCCTGGGCAAGCTGCAGAACGTGGTGAACCAGAACGCCCAGGCCCTGAACACCC
TGGTGAAGCAGCTGAGCAGCAACTTCGGCGCCATCAGCAGCGTGCTGAACGACA
TCCTGAGCAGACTGGACCCCCCTGAGGCCGAGGTGCAGATCGACAGACTGATCA
CCGGCAGACTGCAGAGCCTGCAGACCTACGTGACCCAGCAGCTGATCAGAGCCG
CCGAAATCAGAGCCAGCGCCAATCTGGCCGCCACCAAGATGAGCGAGTGCGTGC

TGGGCCAGAGCAAGAGAGTGGACTTCTGCGGCAAGGGCTACCACCTGATGAGCT
TTCCCCAGAGCGCCCCTCACGGCGTGGTGTTTCTGCACGTGACCTACGTGCCTGC
CCAGGAGAAGAACTTCACCACCGCCCCTGCCATCTGCCACGATGGCAAGGCCCA
CTTCCCTAGAGAGGGCGTGTTCGTGAGCAACGGCACCCACTGGTTCGTGACCCAG
AGAAACTTCTACGAGCCCCAGATCATCACCACCGACAACACCTTCGTGAGCGGC
AACTGCGACGTGGTGATCGGCATCGTGAACAACACCGTGTACGACCCCCTGCAG
CCCGAGCTGGACAGCTTCAAGGAGGAGCTGGACAAGTACTTCAAGAACCACACC
AGCCCCGATGTGGACCTGGGCGATATCAGCGGCATCAATGCCAGCGTGGTGAAC
ATCCAGAAGGAGATCGACCGGCTCAATGAGGTGGCCAAGAACCTGAACGAGAG
CCTGATCGACCTGCAGGAACTGGGCAAATATGAGCAGTACATCAAGTGGCCCTG
GTACATCTGGCTGGGCTTCATCGCCGGCCTGATCGCCATCGTGATGGTGACCATC
ATGCTGTGCTGCATGACCAGCTGCTGCAGCTGCCTGAAGGGCTGCTGCAGCTGCG
GGTCTTGTTGCTAATCTAGAGGGCCCGTTTAAACCCGCTGATCAGCCTCGACTGT
GCCTTCTAGTTGCCAGCCATCTGTTGTTTGCCCCTCCCCCGTGCCTTCCTTGACCC
TGGAAGGTGCCACTCCCACTGTCCTTTCCTAATAAAATGAGGAAATTGCATCGCA
TTGTCTGAGTAGGTGTCATTCTATTCTGGGGGGTGGGGTGGGGCAGGACAGCAA
GGGGGAGGATTGGGAAGACAATAGCAGGCATGCTGGGGATGCGGTGGGCTCTAT
GGCTTCTACTGGGCGGTTTTATGGACAGCAAGCGAACCGGAATTGCCAGCTGGG
GCGCCCTCTGGTAAGGTTGGGAAGCCCTGCAAAGTAAACTGGATGGCTTTCTTGC
CGCCAAGGATCTGATGGCGCAGGGGATCAAGCTCTGATCAAGAGACAGGATGAG
GATCGTTTCGCATGATTGAACAAGATGGATTGCACGCAGGTTCTCCGGCCGCTTG
GGTGGAGAGGCTATTCGGCTATGACTGGGCACAACAGACAATCGGCTGCTCTGA
TGCCGCCGTGTTCCGGCTGTCAGCGCAGGGGCGCCCGGTTCTTTTTGTCAAGACC
GACCTGTCCGGTGCCCTGAATGAACTGCAAGACGAGGCAGCGCGGCTATCGTGG
CTGGCCACGACGGGCGTTCCTTGCGCAGCTGTGCTCGACGTTGTCACTGAAGCGG
GAAGGGACTGGCTGCTATTGGGCGAAGTGCCGGGGCAGGATCTCCTGTCATCTC
ACCTTGCTCCTGCCGAGAAAGTATCCATCATGGCTGATGCAATGCGGCGGCTGCA
TACGCTTGATCCGGCTACCTGCCCATTCGACCACCAAGCGAAACATCGCATCGAG
CGAGCACGTACTCGGATGGAAGCCGGTCTTGTCGATCAGGATGATCTGGACGAA
GAGCATCAGGGGCTCGCGCCAGCCGAACTGTTCGCCAGGCTCAAGGCGAGCATG
CCCGACGGCGAGGATCTCGTCGTGACCCATGGCGATGCCTGCTTGCCGAATATCA
TGGTGGAAAATGGCCGCTTTTCTGGATTCATCGACTGTGGCCGGCTGGGTGTGGC
GGACCGCTATCAGGACATAGCGTTGGCTACCCGTGATATTGCTGAAGAGCTTGGC
GGCGAATGGGCTGACCGCTTCCTCGTGCTTTACGGTATCGCCGCTCCCGATTCGC
AGCGCATCGCCTTCTATCGCCTTCTTGACGAGTTCTTCTGAATTATTAACGCTTAC
AATTTCCTGATGCGGTATTTTCTCCTTACGCATCTGTGCGGTATTTCACACCGCAT
CAGGTGGCACTTTTCGGGGAAATGTGCGCGGAACCCCTATTTGTTTATTTTTCTA
AATACATTCAAATATGTATCCGCTCATGAGACAATAACCCTGATAAATGCTTCAA
TAATAGCACGTGCTAAAACTTCATTTTTAATTTAAAAGGATCTAGGTGAAGATCC
TTTTTGATAATCTCATGACCAAAATCCCTTAACGTGAGTTTTCGTTCCACTGAGCG
TCAGACCCCGTAGAAAAGATCAAAGGATCTTCTTGAGATCCTTTTTTTCTGCGCG
TAATCTGCTGCTTGCAAACAAAAAAACCACCGCTACCAGCGGTGGTTTGTTTGCC
GGATCAAGAGCTACCAACTCTTTTTCCGAAGGTAACTGGCTTCAGCAGAGCGCA
GATACCAAATACTGTTCTTCTAGTGTAGCCGTAGTTAGGCCACCACTTCAAGAAC
TCTGTAGCACCGCCTACATACCTCGCTCTGCTAATCCTGTTACCAGTGGCTGCTGC
CAGTGGCGATAAGTCGTGTCTTACCGGGTTGGACTCAAGACGATAGTTACCGGAT
AAGGCGCAGCGGTCGGGCTGAACGGGGGGTTCGTGCACACAGCCCAGCTTGGAG
CGAACGACCTACACCGAACTGAGATACCTACAGCGTGAGCTATGAGAAAGCGCC
ACGCTTCCCGAAGGGAGAAAGGCGGACAGGTATCCGGTAAGCGGCAGGGTCGG
AACAGGAGAGCGCACGAGGGAGCTTCCAGGGGGAAACGCCTGGTATCTTTATAG

TCCTGTCGGGTTTCGCCACCTCTGACTTGAGCGTCGATTTTTGTGATGCTCGTCAG
GGGGGCGGAGCCTATGGAAAAACGCCAGCAACGCGGCCTTTTTACGGTTCCTGG
CCTTTTGCTGGCCTTTTGCTCACATGTTCTT
